# Linker Flexibility Facilitates Module Exchange in Fungal Hybrid PKS-NRPS Engineering

**DOI:** 10.1371/journal.pone.0161199

**Published:** 2016-08-23

**Authors:** Maria Lund Nielsen, Thomas Isbrandt, Lene Maj Petersen, Uffe Hasbro Mortensen, Mikael Rørdam Andersen, Jakob Blæsbjerg Hoof, Thomas Ostenfeld Larsen

**Affiliations:** Department of Systems Biology, Technical University of Denmark, Søltofts Plads, Kongens Lyngby, Denmark; University of Nebraska-Lincoln, UNITED STATES

## Abstract

Polyketide synthases (PKSs) and nonribosomal peptide synthetases (NRPSs) each give rise to a vast array of complex bioactive molecules with further complexity added by the existence of natural PKS-NRPS fusions. Rational genetic engineering for the production of natural product derivatives is desirable for the purpose of incorporating new functionalities into pre-existing molecules, or for optimization of known bioactivities. We sought to expand the range of natural product diversity by combining modules of PKS-NRPS hybrids from different hosts, hereby producing novel synthetic natural products. We succeeded in the construction of a functional cross-species chimeric PKS-NRPS expressed in *Aspergillus nidulans*. Module swapping of the two PKS-NRPS natural hybrids CcsA from *Aspergillus clavatus* involved in the biosynthesis of cytochalasin E and related Syn2 from rice plant pathogen *Magnaporthe oryzae* lead to production of novel hybrid products, demonstrating that the rational re-design of these fungal natural product enzymes is feasible. We also report the structure of four novel pseudo pre-cytochalasin intermediates, niduclavin and niduporthin along with the chimeric compounds niduchimaeralin A and B, all indicating that PKS-NRPS activity alone is insufficient for proper assembly of the cytochalasin core structure. Future success in the field of biocombinatorial synthesis of hybrid polyketide-nonribosomal peptides relies on the understanding of the fundamental mechanisms of inter-modular polyketide chain transfer. Therefore, we expressed several PKS-NRPS linker-modified variants. Intriguingly, the linker anatomy is less complex than expected, as these variants displayed great tolerance with regards to content and length, showing a hitherto unreported flexibility in PKS-NRPS hybrids, with great potential for synthetic biology-driven biocombinatorial chemistry.

## Introduction

**Fig 1:**
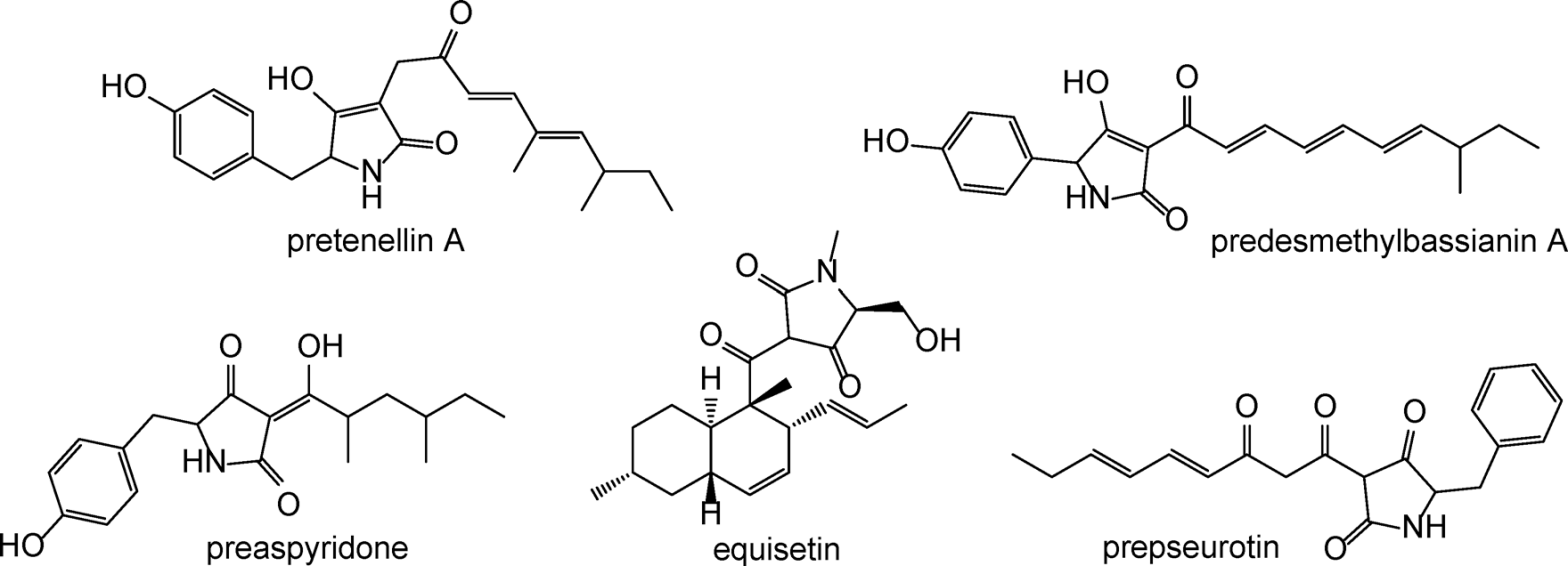
Structures of known PKS-NRPS products [5–9].

Polyketide synthases (PKSs) and nonribosomal synthetases (NRPSs) are among the major biosynthetic enzymes for fungal secondary metabolites, and are responsible for the biosynthesis of numerous medically relevant compounds including statins, mycophenolic acid, cyclosporine, and penicillin [1,2], and within the last ten years, natural fusions of PKSs and NRPSs (PKS-NRPSs) have been described [3,4]. These large modular enzymes consist of a type I iterative highly reducing PKS fused to a single NRPS module. Characteristic of fungal PKS-NRPSs is the lack of a functional enoyl reductase (ER) domain, and most of these enzymes therefore rely on a *trans*-acting ER for production of polyketide-amino acid compounds [4]. Most commonly, the PKS-NRPS hybrid and its cognate ERs are encoded in the same cluster. From several studies, the products of PKS-NRPS hybrids co-expressed with their cognate ER have been revealed (Fig 1).

Fungal PKS-NRPSs have been reported to produce highly bioactive compounds, with some of the most prominent examples being pseurotin, cyclopiazonic acid, fusarin C, and cytochalasins [10–13]. For many years, the most common approach to discover new natural drug leads has relied on screening of a large number of organisms often followed by semi-synthetic modifications for final drug structure optimization [14]. For a more sustainable and economical production of natural product derivatives, synthetic biology offers new alternatives, and recent discoveries have sparked the interest for producing analogs of polyketides, nonribosomal peptides, as well as hybrid compounds using combinatorial biosynthetic approaches. By exploiting the modularity of enzymes involved in secondary metabolism, it has been proved feasible to produce novel synthetic compounds by identifying genetic modules, and combine them as building blocks at the genetic level, the goal being the rational design of novel chimeric proteins with desired catalytic properties [15].

The limited structural information on fungal iterative PKSs is the biggest obstacle towards understanding enzyme programming, and thus, product formation. Several studies have been conducted on natural hybrid-, chimeric- and dissected PKS-NRPSs and successful construction of functional chimeric PKS-NRPSs has been achieved in a few studies. One example is the elegant domain and module swapping involving several PKS-NRPS variants, where Cox and co-workers were able to resurrect the extinct metabolite bassianin, as well as reveal some of the underlying mechanisms for product formation [5,16]. The two enzymes used for these experiments, TenS and DmbS, are involved in the production of tenellin and desmethylbassianin, respectively, and the genes encoding TenS and DmbS (87% sequence identity) both originate from the insect pathogen *Beauveria bassiana*. In another study [8], the PKS module of the aspyridone producing PKS-NRPS (ApdA) and the NRPS module of the PKS-NRPS involved in production of cyclopiazonic acid (CpaS) were expressed as individual proteins in *Saccharomyces cerevisiae*, which led to the incorporation of a tryptophan residue into the aspyridone polyketide backbone. Most of the studies have considered only single module swaps and it has therefore been difficult to determine general mechanistic similarities. Recently, a comprehensive study of PKS-to-NRPS compatibility was conducted by Schmidt and co-workers [9]. They constructed 34 distinct module swaps, and in addition to revealing compelling new information on the programming rules of hybrid PKS-NRPSs, they succeeded in the production of a chimeric PKS-NRPS product. Fusion of the equisetin PKS module (EqiS) with the fusaridione A NRPS module (FsdS), both from *Fusarium heterosporum*, resulted in production of the predicted chimeric compound.

So far, no studies have investigated the importance of the PKS-NRPS inter-modular linker. From the existence of many diverse modular proteins in nature, it has long been known that gene duplications as well as the modular assembly of existing genes is a major source of evolutionary novelty [17]. Multidomain proteins are thought to have evolved by gene duplications or by shuffling of sequences encoding different protein domains. From studying the sequence, it is evident that protein domains and modules are often separated by linker sequences that vary greatly in size, and it is well-known that the properties of these linkers are highly sequence-dependent. Changes in the length and flexibility of the linker can have several implications for protein stability- and folding rates, domain-domain interactions, and enzyme activity [18]. Despite the interest in engineering of compounds of mixed biosynthetic origin in fungi, no studies have thoroughly looked into the role of the inter-modular linker of fungal PKS-NRPS hybrids.

In this work, we chose to apply synthetic biology to cytochalasans due to their wide range of distinctive biological functions [19]. We report successful module swapping between the two PKS-NRPS hybrids CcsA from *A*. *clavatus*, which has been shown to be involved in the biosynthesis of cytochalasin E [20], and the related previously uncharacterized Syn2 from the rice plant pathogen *Magnaporthe oryzae* leading to novel hybrid products heterologously expressed in *A*. *nidulans*. For the first time, we have methodically tested the inter-modular PKS-NRPS linker for its role in the transfer of biosynthetic intermediates. By expression of several linker-modified variants, we demonstrate that these linkers display great tolerance with regards to content and length, showing a hitherto unreported flexibility in PKS-NRPS hybrids, with great potential for synthetic biology-driven biocombinatorial chemistry.

## Results and Discussion

### Co-expression of *ccsA* and *ccsC* in *A*. *nidulans* leads to production of a modified cytochalasin intermediate

**Fig 2:**
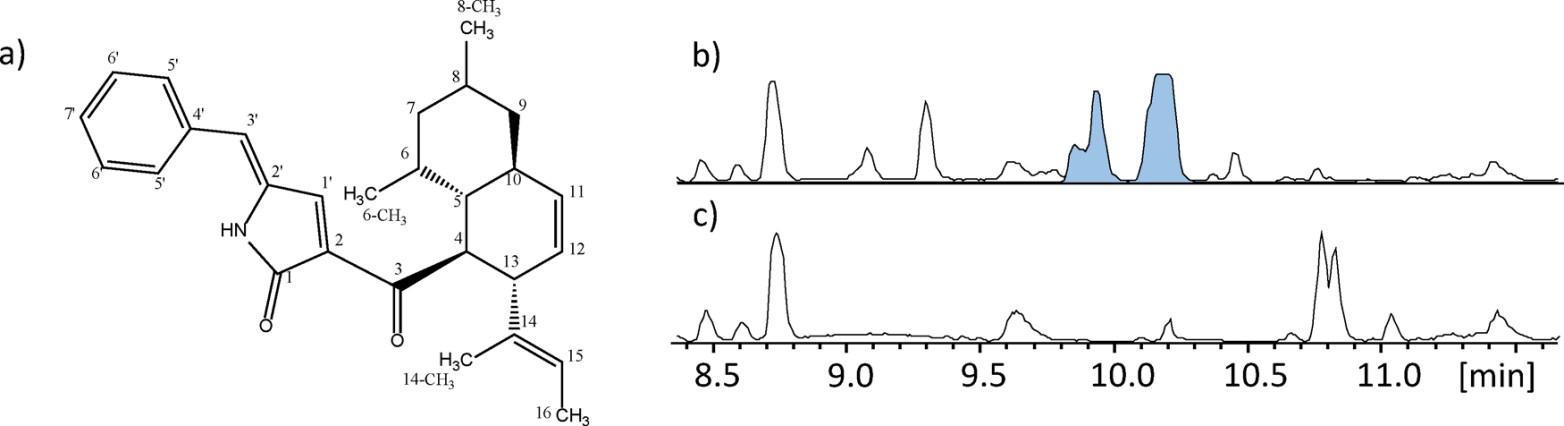
Overexpression of *ccsA* and *ccsC* in *A*. *nidulans* leads to production of niduclavin. A) The structure of niduclavin, elucidated by NMR spectrocopy, B) Base peak chromatogram (BPC) of *A*. *nidulans* extracts, showing production of niduclavin (extracted ion chromatogram (EIC) @ *m/z* 416.2584 highlighted in blue), and c) BPC of reference strain, which displayed no production of niduclavin.

To investigate if the *A*. *clavatus* hybrid PKS-NRPS (ACLA_078660) could be functionally expressed in *A*. *nidulans*, *ccsA* along with the *trans*-acting ER *ccsC* (ACLA_078700) encoded in the same gene cluster, were transformed in a two-step approach into *A*. *nidulans*. The strain was analyzed by ultra-high performance liquid chromatography (UHPLC) coupled with diode array detection (DAD) and high-resolution mass spectrometry (HRMS), and the metabolite profile revealed the appearance of a new major compound with a mass of 415.2585 Da ([M+H]^+^ = 416.2584) corresponding to the elemental composition C_28_H_33_NO_2_. It was found that detection of this product was completely dependent on co-expression with the ER as no products were detected in its absence. We successfully purified the heterologous product of the CcsA/CcsC expressing strain, and NMR structural elucidation (see S1 Dataset) revealed a hybrid polyketide-nonribosomal peptide, consisting of the expected phenylalanine moiety joined to a decalin scaffold, originating from a highly reduced polyketide chain, via a tetramic acid derived lactam (Fig 2). We named this new heterologously expressed hybrid product niduclavin.

**Fig 3:**
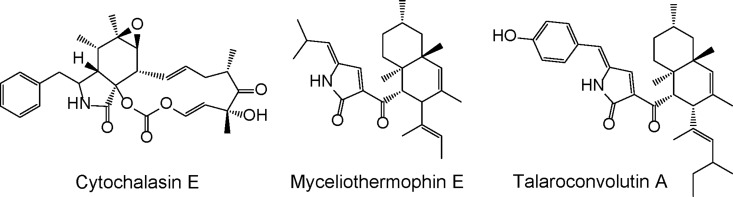
Structures of cytochalasin E, myceliothermophin E, and talaroconvolutin A.

Considering the general structure of cytochalasins such as cytochalasin E (Fig 3), two elements of the structure of niduclavin were unexpected. A double bond was found between the C-2’ and C-3’ position of the phenylalanine side chain, which is a feature that to our knowledge has only been found in talaroconvolutin A, from *Talaromyces convolutes* [21] and myceliothermophin E, from *Myceliophthora thermophile* [22] (Fig 3). Even more interesting, the [4+2]-cyclisation normally encountered in cytochalasin biosynthesis is absent. Instead, a decalin ring system was found, rather than the normally observed 11 membered macrocycle fused to a bicyclic lactam (isoindolone).

We speculate that cross-chemical reactions with endogenous *A*. *nidulans* activities are responsible for the introduction of the double bond. By introducing the additional double bond, additional activation of the dienophile in the α/β-position of the ketone (C-3) would occur, thereby possibly favoring the decalin formation in this position rather than at the tetramic acid moiety present in the *A*. *clavatus* molecule (see S1 Fig). Whether this reaction requires an enzymatic activity is unclear, however the formation of a couple of earlier eluting likely isomeric niduclavin analogues (Fig 2B), suggests that the reaction is non-enzymatic. In nature, there are numerous examples of PKS- and PKS-NRPS products containing decalin ring systems, *e*.*g*. lovastatin, equisetin, talaroconvolutin, and codinaeopsin [21,23–25]. Since the decalin ring system is not present in the cytochalasins, it seems likely that formation of the isoindolone moiety in *A*. *clavatus* is enzyme catalyzed.

We searched the literature for possible candidate enzymes in *A*. *nidulans* that could be responsible for the introduction of the double bond between the C-2’ and C-3’ position of the phenylalanine side chain. The dioxygenase AsqJ (ANID_09227) from *A*. *nidulans* has previously been shown to introduce a double bond in the same position of a phenylalanine moiety of an intermediate in the cyclopenin/4’-methoxycyclopenin biosynthetic pathway [26]. However, deletion of *asqJ* did not result in any changes of the final product of CcsA and CcsC. As an alternative approach to create a proper cytochalasin intermediate, the two genes were also expressed in *A*. *niger*. Yet, expression of *ccsA* and *ccsC* in *A*. *niger* also lead to production of niduclavin (data not shown). The results point to the potential problems with heterologous expression, meaning we cannot by default assume that a product or intermediate produced in a non-native host is identical to the compound produced in the native organism.

In addition to niduclavin, the extract of the CcsA/CcsC expressing strain contained four additional compounds, including one with the mass 440.3188 Da. This mass is identical to the mass of the cytochalasin precursor product that was described by Fujii *el al*. [27] in *A*. *oryzae*, and we speculate that our compound is identical to theirs. Our results and the results of Fujii *et al*. suggest that formation of the characteristic cytochalasin isoindolone and macrocycle moieties in *A*. *clavatus* requires a chemoselective functionality in order to direct the rearrangement reaction towards the macrocyclic pre-cytochalasin product. The existence of this type of activity is supported by a study by Kasahara *et al*. where the biosynthesis of solanapyrone from *Alternaria solani* was investigated [28]. They identified a flavin-dependent oxidase catalyzing an oxidation and mediating a cycloaddition. To our knowledge, no equivalent activities have been found in the *A*. *clavatus* genome. Recently, Klas *et al*. [29] called into question the existence of true Diels Alderases, and it appears that enzymes catalyzing [4+2]-cycloadditions in most cases serve as multifunctional enzymes, *e*.*g*. oxidations as seen in the case of solanapyrone biosynthesis. If the [4+2]-cycloaddition in cytochalasin E is a secondary activity of another enzyme, it is conceivable that the formation of the cytochalasin isoindolone moiety is mediated by one of the tailoring enzymes encoded in the *ccs* cluster. However, cytochalasin biosynthesis in *A*. *nidulans* is believed to be hampered by the formation of the decalin ring system, hereby preventing the formation of the expected isoindolone moiety.

### The Syn2 PKS-NRPS hybrid produces a novel polyketide-nonribosomal peptide intermediate

Based on previous studies, we hypothesized that construction of active chimeric PKS-NRPSs would be dependent on the degree of sequence identity between the recombined modules [5,9,16]. A BLAST search identified a putative PKS-NRPS from *Magnaporthe oryzae* (CAG_28798) with 68% amino acid sequence similarity as the closest homolog to *A*. *clavatus* CcsA (52% identity). The gene, known as *syn2*, is encoded in a previously described gene cluster where another PKS-NRPS hybrid (*ace1*) is also found [30]. Associated genes encoding ERs are predicted for both *ace1* and *syn2* (*rap1* and *rap2*, respectively). Special attention has previously been devoted to this gene cluster because it was shown that the product of the Ace1 pathway is an avirulence factor and is recognized in *Pi33* rice cultivars making them resistant to fungal infection [31]. It was later demonstrated that genes of the Ace1 gene cluster were expressed exclusively in the appressorium during infection of the host plant, although, deletion of *syn2* did not affect avirulence [30].

The only *M*. *oryzae* strain having a publically available genome sequence is the laboratory strain 70–15 [32], which is derived from the wild-type strain Guy-11 through several backcrossings [33]. However, it has been reported that the *syn2* allele in strain 70–15 is inactive, due to an early stop codon from an insertion of a single base pair [30]. The sequence of *syn2* from the wild-type strain Guy-11 has been published (CAG28798) [31], and this allele does not contain this stop codon [30]. To analyze the function of Syn2, we purchased the coding sequences of the Guy-11 *syn2* and its corresponding ER *rap2* (MGG_08380). Seven introns are annotated in the publicly available sequence of Guy-11 *syn2* although only five are predicted using the Augustus gene prediction software (http://bioinf.uni-greifswald.de/augustus/). By including the two putative introns (nucleotides 7962 to 8082 and 8145 to 8323) as part of the coding sequence, a complete amino acid alignment of *syn2* and *ccsA* was possible. To support both scenarios we therefore chose to include these two sequences as part of the coding sequence in the purchased gene.

**Fig 4:**
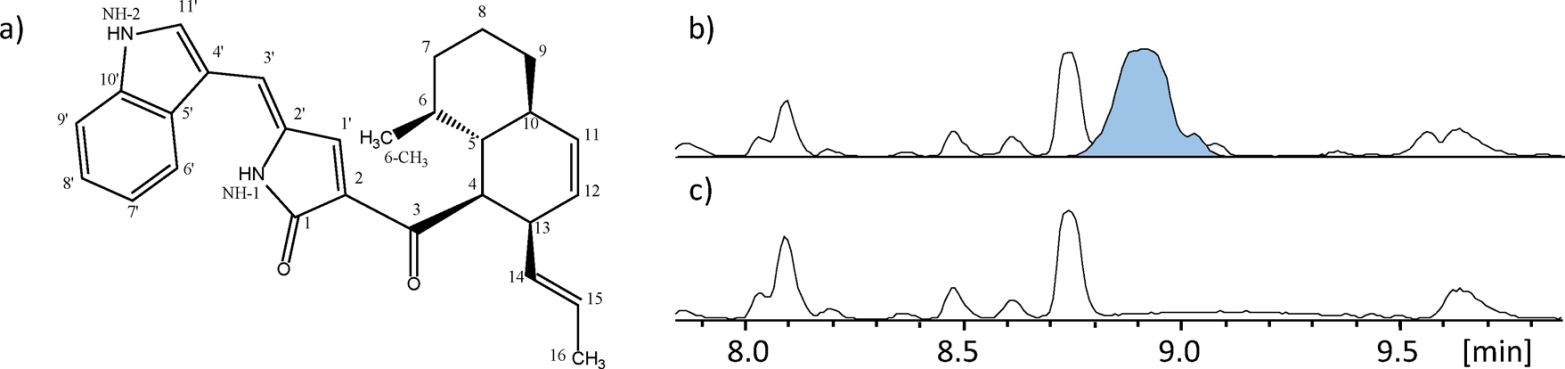
Overexpression of *syn2* and *rap2* in *A*. *nidulans* leads to production of niduporthin. A) The structure of niduporthin, elucidated by NMR spectrocopy, B) BPC of *A*. *nidulans* extracts, showing production of niduporthin (EIC @ *m/z* 427.2380 highlighted in blue), and C) BPC of reference strain, which displayed no production of niduporthin.

Analogous to the integration and expression of *ccsA* and *ccsC*, *syn2* and *rap2* were transformed in two steps into *A*. *nidulans* and the metabolite profile of the strain was analyzed by UHPLC-DAD-HRMS. Expression of the two genes resulted in the production of a compound with a mass of 426.2307 Da ([M+H]^+^ = 427.2380]) corresponding to the formula C_28_H_30_N_2_O_2_. Again, it was found that co-expression with the ER was required for detection of a product. The Syn2/Rap2 product was purified and a structure with high resemblance to niduclavin was determined by NMR (Fig 4; S1 Dataset). The Syn2/Rap2 product expressed in *A*. *nidulans* was named niduporthin. The compound also contained a highly reduced polyketide chain, decalin rings and a nitrogen-containing tetramic acid unit. In conclusion, we wanted to establish whether the two annotated introns that were included in the purchased gene of *syn2* do in fact constitute part of the coding sequence. Therefore, RNA was purified from the *syn2*/*rap2* strain followed by cDNA synthesis. Sequencing of *syn2* cDNA in the regions of the annotated introns confirmed that *A*. *nidulans* does not splice these two sequences suggesting that they do indeed constitute part of the coding sequence.

The polyketide backbone moiety of niduporthin revealed that the two PKS modules of CcsA and Syn2 perform the same number of iterative elongation steps (seven). However, the methyltransferase (MT) domain of Syn2 attaches only one methyl group to the niduporthin backbone in contrast to the three methyl groups added by the MT domain in CcsA. Furthermore, the polyketide is connected to a tryptophan residue instead of phenylalanine demonstrating different adenylation (A) domain specificities. As for niduclavin, a double bond is found between C-2’ and C-3’ of niduporthin, which again supports our hypothesis of cross-chemical interactions by endogenous enzyme(s) in *A*. *nidulans*.

Recently, a product of the Ace1 gene cluster was identified by co-expression of Ace1 with its cognate ER Rap1 in *A*. *oryzae* [34]. This experiment revealed a highly reduced nonaketide backbone conjugated to a tyrosine moiety, but in contrast to niduclavin, niduporthin, and many other previously described PKS-NRPS products, the compound did not contain a tetramic acid moiety and was instead identified as a linear polyketide where the terminal carboxylic acid group was reduced to an alcohol. It was suggested that this compound was unlikely to be the direct precursor of the final product, but interestingly, the compound was similar to the CcsA/CcsC product described by Oikawa and co-workers [27,35]. In both studies, *A*. *oryzae* was used as expression hosts. Thus, it seems that *A*. *oryzae* as well as *A*. *nidulans* modifies the PKS-NRPS products, albeit in different manners.

Hence, niduporthin is the second purified product of the Ace1 gene cluster, and although deletion of *syn2* was shown to have no significant effect on avirulence or plant host infection, the final product of the syn2 pathway could still play an accessory function during the infection process. Khaldi *et al*. [36] has proposed that the Ace1/Syn2 gene cluster arose through a partial tandem duplication, and that subsequently, five of the genes, including Syn2, were transferred to an ancestor of *A*. *clavatus* by horizontal gene transfer. Due to structural similarities to the CcsA/CcsC product presented by Fujii *et al*., Cox and co-workers proposed that the Ace1 pathway constitute a “cytochalasan-like” biosynthetic pathway [34]. As shown by Khaldi et al [36] the Syn2-associated part of the cluster is even more closely related to the *ccs* gene cluster, thus suggesting that the product of the syn2 pathway in *M*. *oryzae* is also likely to be a cytochalasan-type of compound. The structure of niduporthin presented in this work, with its close resemblance to the structure of niduclavin, further corroborates this hypothesis.

### Investigating NRPS A domain substrate specificity

Exchange of amino acids in fungal polyketide-nonribosomal peptide products have been attempted a number of times by swapping entire NRPS modules [9,16,37]. An alternative and perhaps easier approach when working with these large genes, could be to simply change the A domain specificity by introduction of point mutations. Several models for prediction of the specificity-conferring amino acids of bacterial and fungal NRPS A domains have been published [37–41]. The specificity of bacterial NRPS A domains is well-established; however, the identity of the specificity-conferring amino acids of fungal A domains is much less characterized, which is perhaps due to a more complex mechanism of amino acid selectivity compared to bacterial A domains.

To investigate the specificities of the A domains of CcsA and Syn2, we used the “NRPSpredictor2” web server (http://nrps.informatik.uni-tuebingen.de/) [41,42] to define the signature amino acid residues that are predicted to determine the identity of the amino acid incorporated by the NRPS module. Despite the two A domains sharing only around 48% sequence identity, the ten amino acids predicted to line the active-site binding pocket of CcsA (DMS**E**VG**C**FCK) and Syn2 (DMS**S**VG**G**FCK) vary only in two positions between the enzymes, suggesting that these two residues would explain the observed difference in amino acid specificity. This observation therefore made CcsA and Syn2 an ideal case for studying the quality of the prediction, and of the A domain specificity at the primary structure level. The CcsA A domain uses phenylalanine as substrate while Syn2 uses the larger tryptophan. We attempted to switch the A domain substrate specificities by interchanging the amino acids at these two positions. For each of the enzymes, three strains were constructed; two carrying a single point mutations and one carrying the double mutation.

**Fig 5:**
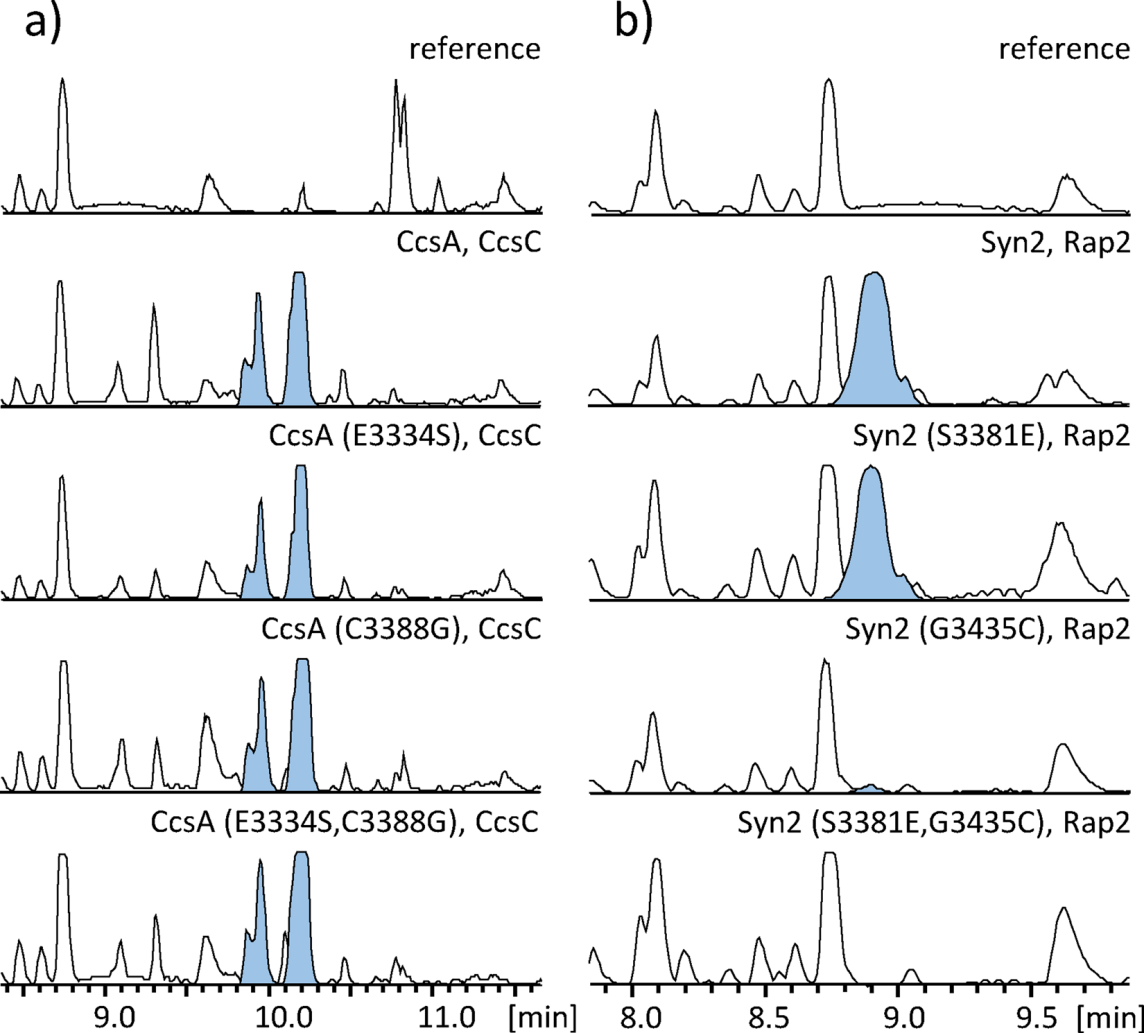
The effects of point mutations in the CcsA- and Syn2 A domains on product formation. The chromatograms show BPCs of *A*. *nidulans* extracts expressing various single- and double mutations in the NRPS A domain as specified above the traces. EICs for niduclavin and niduporthin are highlighted in blue. A) Expression of CcsA containing single- and double point mutations (E3334S and C3388G). B) Expression of Syn2 containing single- and double point mutations (S3381E and G3435C). Only niduporthin-production by Syn2 was affected by introduction of the mutations.

For CcsA, no significant effect of any point mutations was observed on the production of niduclavin, and we were unable to detect any tryptophan-incorporation in niduclavin in the extract (Fig 5A). In contrast, Syn2-(G3435C) produced only trace amounts of niduporthin, whereas the serine-to-glycine mutation in *syn2* had no effect on niduporthin production. Interestingly, the two mutations combined in Syn2 resulted in a complete loss of product (Fig 5B). In order to facilitate comparisons across strains containing different point mutations, we performed a Southern blot analysis of all these strains to investigate whether the gene copy number could account for the observed differences in the level of product formation. Southern blot analysis indicated that the strain containing Syn2-(G3435C) carried an extra copy of the gene (see S2 Fig). Despite this extra copy, the production of niduporthin was very low compared to unmodified Syn2.

The decrease in product formation observed for Syn2 could perhaps be explained by substitution of smaller amino acids (Ser and Gly) for more bulky amino acids (Glu and Cys), thereby causing a steric clash in the active site, and rendering the enzyme unable to accommodate the more bulky tryptophan substrate. In this scenario, the opposite mutations introduced in CcsA would result in a more spacious binding pocket. It was shown that this change has no effect on niduclavin production but also did not lead to incorporation of tryptophan in place of phenylalanine. The results seem to indicate that the two amino acids are indeed located in the active site binding pocket. However, switching specificities was not achieved, which could suggest that amino acid substrate selection does not solely or directly involve the 10 amino acids predicted by NRPSpredicter2, and that other mechanisms control A domain substrate selectivity. Overall, the rules governing amino acid substrate specificity in fungal NRPSs are likely more complex than for bacterial systems, and it appears that the 10 amino acids predicted for fungal A domains are either not valid, or not sufficient to explain amino acid selectivity.

### The CcsA intermodular region shows high tolerance for length and content

One crucial feature for construction of functional chimeric PKS-NRPSs is to understand the transfer mechanisms of polyketide intermediates to the NRPS module. These mechanisms may include protein-protein interactions *i*.*e*. compatibility of non-cognate modules along with the mechanism of NRPS substrate selectivity. We considered another mechanism involving linker-mediated polyketide transfer. When examining the domain architecture of CcsA, we observed a region between the acetyl carrier protein (ACP) domain of the PKS module and the condensation (C) domain of the NRPS module containing a stretch of approximately 150 amino acid residues. A BLAST search of the primary sequence of CcsA indicated the presence of an inter-modular region with no homology to other PKS-NRPSs, predicted to display no intrinsic enzymatic activity. We considered an influence of this linker in controlling polyketide chain transfer between the PKS- and NRPS modules, and transfer efficiency would thus be dependent on the length- and amino acid sequence of the linker.

Consequently, we investigated the potential influence of linker composition on products formation by constructing several linker-modified variants of CcsA and Syn2. For linker-swapping, three linkers from different PKS-NRPS homologues were selected (Table 1). The linkers were defined by a combination of alignments and domain predictions, varying in length and sharing no significant sequence similarity to CcsA in the linker region. The linker sequences were PCR amplified and fused to the PKS- and NRPS sequences of *ccsA*, thereby replacing the native linker sequence. The resulting plasmids were transformed into an *A*. *nidulans ccsC* background strain, and the verified strains were analyzed by UHPLC-DAD-HRMS. The results showed that all linker variants displayed niduclavin production comparable to unmodified CcsA, and the BPCs of all linker-modified variants were comparable to Fig 2B. Thus, no effects of the linker exchange were detected (see S3 Fig). Additionally, four linker-truncated variants of CcsA were constructed (Table 1), however, no effects were observed for any truncations and niduclavin production was retained for all variants. Surprisingly, even the complete removal of the linker, replaced only by a short flexible GSG linker, had no observable effect on product formation. This apparent redundancy of the linker was also tested for Syn2, in which the 163 amino acid linker was replaced with a GSG linker, also showing no significant influence on niduporthin production (see S3 Fig). Finally, a globular protein in the form of red fluorescent protein (RFP) was placed in between the two modules; in one construct situated within the linker, while replacing the linker in another construct. The functionality of the RFP fluorophore was confirmed by fluorescence microscopy (data not shown), and the strains were analyzed by UHPLC-DAD-HRMS. Again, niduclavin production was fully retained. In summary, the results imply that no selection for the linker content exists.

**Table 1:** Linker-modified variants of CcsA and Syn2^a^.

Strain	Linker modification^*b*^	Linker length (amino acid)
*CcsA* WT	None	150
*CcsA*-CAC	Linker swap–*A. nidulans* AN8412	105
*CcsA*-CEC	Linker swap–*A. clavatus* ACLA_023380	69
*CcsA*-CMC	Linker swap–*M. oryzae* CAG28798	163
*CcsA*-LΔ150	Deletion in the central part of the linker	100
*CcsA*-LΔ225up	Deletion of the N-terminal end of the linker	75
*CcsAL*Δ225dw	Deletion of the C-terminal end of the linker	75
*CcsA*-L-GSG	Substitution of the linker for Gly-Ser-Gly	3
*Syn2*-L-GSG	Substitution of the linker for Gly-Ser-Gly	3
*CcsA*-RFPlink1	Insertion of RFP in the linker	373
*CcsA*-RFPlink2	Substitution of the linker for RFP	235

^a^ See also S3 Fig.

^b^ PKS- and NRPS domains were predicted using the NCBI Conserved Domain Database [43] and the linker was defined between the ACP domain and the condensation domain at positions 2487–2636 (amino acid sequence).

The apparent tolerance for the length of the linker prompted us to test whether niduclavin production could be retained when the two modules were expressed as individual proteins. The dissected PKS- and NRPS modules were co-expressed with the ER CcsC in *A*. *nidulans* and analyzed by UHPLC-DAD-HRMS. The strain did not yield any niduclavin. Compartmentalization of one of the modules could account for the lack of niduclavin production. Therefore, the localizations of the PKS- and NRPS modules were investigated by a C-terminal tagging with RFP and mCitrine, respectively. Both proteins, however, appeared to localize to the cytoplasm (see S4 Fig). This result was surprising since it was previously shown that the activity of the aspyridone PKS-NRPS ApdA could be reconstituted *in vitro* when the two modules were expressed as stand-alone enzymes [8]. However, our results suggest that the primary function of the linker simply is to keep the two modules in close proximity. Furthermore, if PKS-NRPS module-module interactions play a significant role in chain transfer, it seems likely that introduction of a globular protein between the two modules would hamper these interactions. Unexpectedly though, when the linker was replaced by RFP no observable effect on niduclavin production was detected. This could suggest that protein-protein interactions, in fact, do not play an essential role in chain transfer–a conclusion that is in line with the results of Schmidt and co-workers, who showed that the criteria for successful amidation of polyketides are beyond simple ACP-to-C domain interactions [9]. Our results therefore suggest that polyketide substrate recognition by the NRPS module could be the key factor to be considered in construction of chimeric PKS-NRPSs. It also suggests that recombination of PKS-NRPS hybrids *in vivo* and between species may be very flexible, facilitating formation of new spontaneous PKS-NRPSs.

### Module swapping of CcsA and Syn2 results in functional chimeric PKS-NRPSs

**Fig 6:**
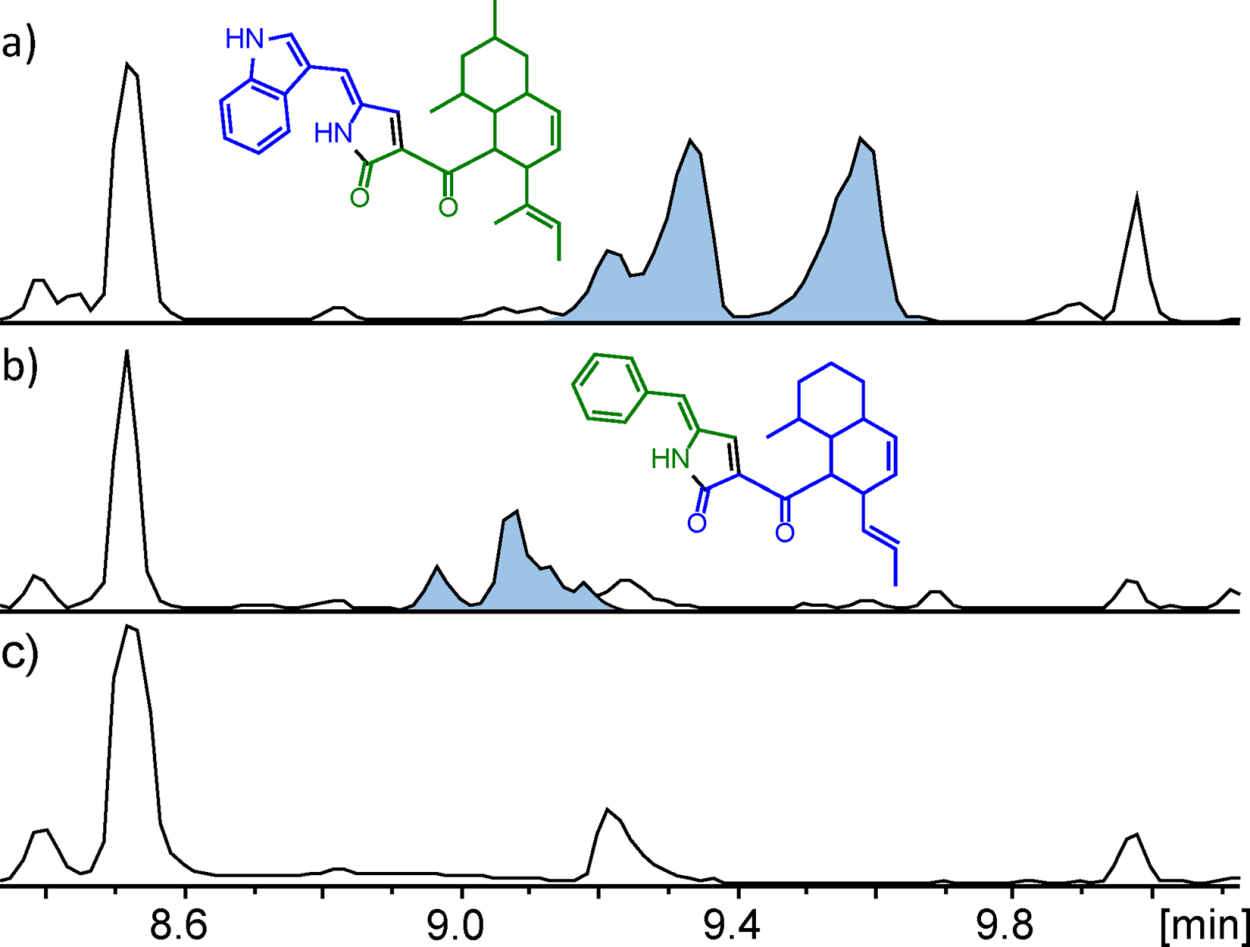
Analysis of chimeric variants of PKS-NRPSs CcsA and Syn2. The chromatograms show BPCs of *A*. *nidulans* extracts expressing PKS-NRPS hybrid compounds. EICs of the products are highlighted in blue along with structures of the predicted compounds: A) Expression of chimeric *ccsA-syn2* leads to production of niduchimaeralin A (m/z 455.2693). B) Expression of chimeric *syn2-ccsA* leads to production of niduchimaeralin B (m/z 388.2271). c) Reference strain.

The linker analysis showed high flexibility and it showed that our definition of PKS and NRPS modules’ respective start and ending appeared correct. This allowed us systematically to fuse PKS and NRPS modules from different species to investigate formation of new chimeric hybrid products. We set out to fuse the PKS module of the *A*. *clavatus ccsA* with the NRPS module of the *M*. *oryzae syn2*. To ensure functionality and to provide information on the optimal site for linkage of the two heterologous modules, six variants of the *ccsA-syn2* combination were constructed (see S5 Fig). Among the six *ccsA-syn2* variants, one was joined in the center of the ACP domain to form a hybrid *ccsA-syn2* ACP domain, while another was joined immediately downstream of the ketoreductase (KR) domain. The latter construct was designed to eliminate effects of protein-protein interactions, a strategy also applied by Schmidt and co-workers [9]. All variants were expressed in *A*. *nidulans* along with the ER *ccsC*, and the resulting strains were analyzed by UHPLC-DAD-HRMS. Strikingly, all six variants produced a compound of the mass 454.2620 Da ([M+H]^+^ = 455.2693) corresponding to an elementary composition of C_30_H_34_N_2_O_2_. Indeed, this corresponded exactly to a compound with the polyketide moiety of niduclavin and the tryptophan residue found in niduporthin. Similar to niduclavin and niduporthin, several isomeric compounds were detected in the extracts. The structure of this chimeric compound (named niduchimaeralin A) could be tentatively identified based on tandem MS analysis (see S1 Dataset), since it had a very similar fragmentation pattern to that of niduclavin, including detections of major fragment ions at *m/*z 203 and *m/z* 109, strongly indicating that the two compounds have identical decalin-containing polyketide backbones (Fig 6A). The finding that all six variants are active further supports our previous finding that the linker is highly flexible with regards to length and composition. This also applies to linking heterologous PKS-NRPS fusions, since joining of non-cognate modules lead to the introduction of a non-cognate linker for one of the modules, which in this case lead to a functional enzyme.

To address whether any of the CcsA-Syn2 chimeras were particularly efficient in product formation and to ensure proper comparison of the strains, integration in the intended locus was investigated by Southern blot analysis. This confirmed that all strains had only a single copy of the chimeric gene in the genome and that it had been integrated in the intended integration locus (S2 Fig). This allowed a rough comparison of niduchimaeralin A production for the six CcsA-Syn2 variants based on the relative peak intensities in the base peak chromatograms (S5 Fig). The chimera joined in the center of the linker appeared to display the highest production suggesting that this linkage was the least disruptive of the six. Comparably, production was only slightly lower when the two modules were joined in the middle of the ACP domain, or in the early part of the condensation domain. This is perhaps not surprising since CcsA and Syn2 display conservancy in these regions. On the other hand, linking the two modules immediately downstream of the linker had a substantial negative effect on niduchimaeralin A production. Similarly, joining the two modules downstream of the KR domain led to a loss of product formation (S5 Fig). Effects of protein-protein interactions between non-cognate ACP- and condensation domains were eliminated, and hence, the loss of niduchimaeralin A production must be attributed to unfavorable interactions between the Syn2 ACP domain and the remaining CcsA PKS domains.

The reciprocal chimeric swap where the PKS module of Syn2 was fused to the NRPS module of CcsA was also constructed. The *syn2-ccsA* chimeric gene was transformed into *A*. *nidulans* and co-expressed with the *M*. *oryzae* ER Rap2. Since fusion in the middle of the linker appeared the least disruptive for the CcsA-Syn2 fusion, the Syn2-CcsA chimera was fused in this manner. The product of a functional Syn2-CcsA PKA-NRPS chimera would be expected to contain the PK moiety of Syn2 and the NRP moiety of CcsA. The constructed strain was analyzed by UHPLC-DAD-HRMS, and indeed the presence of at least three isomeric compounds with the expected mass of 387.2198 Da ([M+H]^+^ = 388.2271) was detected in the extract. Again, this indicates that the decalin ring formation is non-enzymatic. Similar to niduchimeraelin A, tandem MS fragmentation analysis clearly indicated that the structure of the Syn2-CcsA chimeric product, named niduchimaeralin B, contained the polyketide backbone of niduporthin that had likely formed a decalin ring system, fused to a phenylalanine residue of niduclavin (Fig 6B). This was evident from detection of major fragment ions at *m/z* 175 and *m/z* 95 (see S1 Dataset).

The lack of selectivity for the length and content of the linker suggests that protein-protein interactions are not essential for successful amino acid incorporation. This is also in accordance with the findings of Kakule *et al* [9], who constructed a chimeric LovB-EqxS PKS-NRPS where the ACP domain of EqxS was retained. Despite the expected compatibility at the interface between the two modules, the combination did not yield a hybrid polyketide-nonribosomal peptide compound. Consequently, they also hypothesized that C domain substrate selectivity overrules protein-protein interaction.

We speculate that the successful swapping of CcsA and Syn2 modules obtained in this study was achieved, not because of protein structure compatibility, but rather because the products of the two polyketide intermediates are structurally similar. Structural similarity would increase the likelihood that PKS intermediates will be recognized by non-cognate condensation domains and in the case of CcsA and Syn2 will lead to amidation of the polyketide backbone. Our hypothesis is supported by the fact that Syn2 and CcsA do not share overwhelming sequence identity (52%), and yet the polyketide intermediates vary only with two methylations of the backbone. If polyketide chain transfer is indeed determined primarily by substrate recognition by the NRPS C domain, and by proximity provided by the inter-modular linker, recombination of even more distantly related PKS-NRPSs should be achievable given that their respective polyketide intermediates are structurally similar.

## Materials and Methods

### Strains, genomic DNA and media

A list of all the strains used and produced in this study is provided in S1 Table. *Aspergillus nidulans* strain IBT 29539 (*argB2*, *pyrG89*, *veA1*, *nkuAΔ*)—referred to as NID1—was used for heterologous production of niduclavin and niduporthin. *A*. *clavatus* genomic DNA was obtained from strain IBT 12364 (NRRL 1) and was extracted using the FastDNA^TM^ SPIN Kit for Soil DNA extraction (MP Biomedicals, USA). Coding sequence of *M*. *oryzae* genes *syn2* and *rap2* were purchased from GenScript USA. *Escherichia coli* strain DH5α was used for plasmid propagation.

*Aspergillus* solid and liquid minimal medium (MM) and transformation medium (TM) was supplemented when necessary and according to strain genotypes with 4 mM L-arginine, 10 mM uridine, 10 mM uracil, and 1.3 mg/ml 5-fluoroorotic acid (5-FOA), and was prepared as described by Nødvig *et al*. [44]. *E*. *coli* DH5α was cultivated in Luria-Bertani (LB) medium consisting of 10 g/l tryptone (Bacto), 5 g/l yeast extract (Bacto), and 10 g/l NaCl (pH 7.0). LB medium was supplemented with 100 µg/ml ampicillin. All solvent used was of HPLC grade, and H_2_O was purified and deionized by a Millipore system through a 0.22 µm membrane filter (MQ H_2_O).

### Vector- and strain construction

All primers (Integrated DNA Technology, Belgium) used in this study are listed in S2 Table. All PCR fragments were generated using the PfuX7 polymerase [45]. All vectors were constructed by Uracil-Specific Excision Reagent (USER) fusion of PCR fragments into compatible vectors [46]. Genes encoding PKS-NRPS hybrids and ERs were cloned into plasmids (pU2115) designed for overexpression by integration into specific targeting sites in the *A*. *nidulans* genome [47]. The plasmids contain a *Pac*I/*Nt*.*Bbv*CI USER cassette, the constitutive promoter P*gpdA*, the T*trpC* terminator, *A*. *nidulans* gene targeting sequences and *A*. *fumigatus pyrG* flanked by direct repeats for selection and counter selection in *A*. *nidulans*. For plasmid propagation in *E*. *coli* the plasmids also contained the *E*. *coli* ampicillin resistance gene and the origin of replication. The plasmid for deletion of *asqJ* was constructed by introduction of the up- and downstream sequences on each side of the *pyrG* marker. All plasmids were purified using the GenElute^TM^ Plasmid Miniprep Kit (Sigma-Aldrich), and subsequently verified by restriction analysis. For all strains not producing any metabolites, the sequence of the transformed genes were confirmed by sequencing (StarSEQ, Germany) to exclude simple coding errors as reason for not functioning. RNA was purified using the RNeasy Plus Mini Kit from Qiagen, and cDNA was prepared using the Maxima H Minus First Strand cDNA Synthesis Kit from Thermo Scientific^TM^. All vectors were linearized with *Swa*I (New England Biolabs) prior to transformation according to manufacturer’s instructions. *A*. *nidulans* protoplastation, transformation and rigorous strain validation was performed as described by Nødvig *et al*. [44]. For successive integration of genes, strains were plated on MM supplemented with uridine, uracil, and 5-FOA for counter selection of the *pyrG* marker. The Southern blot protocol is provided in S1 Protocol and the fluorescence microscopy protocol is provided in S2 Protocol.

### Chemical analysis

Strains of *A*. *nidulans* were cultivated at 37°C for 6 days on solid MM with the necessary supplements. Plug extractions were performed as described in Smedsgaard, 1997 [48]. The samples were analyzed on a maXis 3G orthogonal acceleration quadrupole time-of-flight mass spectrometer (Bruker Daltonics) equipped with an electrospray ionization (ESI) source and connected to an Ultimate 3000 UHPLC system (Dionex), equipped with a Kinetex 2.6 µm C18, 100mm x 2.1 mm column (Phenomenex). The method applied was described by Holm *et al*. [49].

Tandem MS experiments were done on an Agilent Infinity 1290 UHPLC system (Agilent Technologies, Santa Clara, CA, USA) equipped with a diode array detector. Separation was obtained on an Agilent Poroshell 120 phenyl-hexyl column (2.1 × 250 mm, 2.7 μm) with a linear gradient consisting of water (A) and acetonitrile (B) both buffered with 20 mM formic acid, starting at 10% B and increased to 100% in 15 min where it was held for 2 min, returned to 10% in 0.1 min and remaining for 3 min (0.35 mL/min, 60°C). MS detection was performed in positive mode on an Agilent 6545 QTOF MS equipped with Agilent Dual Jet Stream electrospray ion source with a drying gas temperature of 250°C, gas flow of 8 L/min, sheath gas temperature of 300°C and flow of 12 L/min. Capillary voltage was set to 4000 V and nozzle voltage to 500 V. Mass spectra were recorded at 10, 20 and 40 eV as centroid data for *m*/*z* 85–1700 in MS mode and *m*/*z* 30–1700 in MS/MS mode, with an acquisition rate of 10 spectra/s. For MS^3^, the fragmentor voltage was increased from 120 V to 200 V, and the desired m/z’s (175 and 203) were selected for auto MS/MS. Lock mass solution in 70:30 methanol:water was infused in the second sprayer using an extra LC pump at a flow of 15 μL/min using a 1:100 splitter. The solution contained 1 μM tributylamine (Sigma-Aldrich) and 10 μM Hexakis(2,2,3,3-tetrafluoropropoxy)phosphazene (Apollo Scientific Ltd., Cheshire, UK) as lock masses. The [M + H]^+^ ions (*m*/*z* 186.2216 and 922.0098 respectively) of both compounds was used. Descriptions of the MS/MS fragmentation patterns of niduclavin, niduporthin, and niduchimaeralin A and B are provided in S1 Text, and for MS/MS data see S1 Dataset.

### Purification of Metabolites

For large-scale extracts, strains were cultivated on 6 x 500 mL semi-liquid MM (0.2% agar) at 37°C for 7 days. Extractions were done by separating the mycelium from the media and extracting two times with ethyl acetate (EtOAc); first for one hour with sonication, and second for 12 hrs without sonication. The combined EtOAc phases were dried using a rotary evaporator.

*Niduclavin*: The extract from the large scale extraction consisting of 0.12 g was adsorbed onto Diol material and dried before packing on a 10 g (~16 mL) SNAP column (Biotage, Uppsala, Sweden) with Diol material. The extract was then fractionated on an Isolera One flash purification system (Biotage) using seven steps of heptane-dichloromethane (DCM)-EtOAc-methanol (MeOH). Fractions were automatically collected one CV at a time. The DCM fractions were subjected to further purification on a semi-preparative HPLC, a Waters 600 Controller with a 996 photodiode array detector (Waters, Milford, MA, USA). This was achieved using a Luna II C18 column (250 × 10 mm, 5 μm, Phenomenex) and 50:50% ACN/H_2_O isocratic elution for 5 minutes before increasing to 100% ACN in 15 minutes. The flowrate used was 5 mL/min and 50 ppm TFA of HPLC grade was added to ACN and MQ H_2_O. HRMS analysis of the pure compound gave a mass-to-charge ratio of 416.2584, corresponding to a molecular formula of C_28_H_33_NO_2_ (DBE = 13) (calculated for 416.2584, Δ 0 ppm). The yellow amorphous solid displayed UV absorbance at 242 nm and 373 nm (H_2_O/MeCN).

*Niduporthin*: The crude extract (1.2 g) was adsorbed onto C18 material and dried, followed by packing on a 50 g (~66 mL) SNAP column (Biotage) with C18 material. Fractionation was done on an Isolera One flash purification system (Biotage) using a linear MeOH/H_2_O-gradient from 0 to 100% MeOH over 32 column volumes. Collection was done automatically using the UV signals at 254 nm and 400 nm with a threshold of 20 mAU. The fractions were analysed by UHPLC-DAD-QTOFMS and the ones containing the desired compound were purified further using the same semi-preparative HPLC system (Waters) and column (Phenomenex) as for niduclavin. The method used was an ACN/H_2_O (50 ppm TFA) gradient starting at 65% ACN, increasing to 88% ACN over 8 minutes. Isocratic elution at 88% ACN was done for 8 minutes followed by increasing to 100% ACN over 4 minutes. Analysis by HRMS gave a mass-to-charge of 427.2386, corresponding to a molecular formula of C_28_H_30_N_2_O_2_ (DBE = 15) (calculated for 427.2385, Δ -0.2 ppm). The pure compound was a dark red amorphous solid with UV absorption at 228 nm, 270 nm, 286 nm, and 455 nm.

The total yield of niduclavin was 1.1 mg whereas the yield of niduporthin was 23.5 mg. However, it must be noted that niduclavin was purified from a strain carrying the *ccsA* hybrid gene in a different expression site, and we assume a similar yield had niduclavin been purified from the strain carrying *ccsA* in the same expression site as *syn2*.

### NMR

All spectra were recorded on a Bruker Avance 800 MHz spectrometer located at the Danish Instrument Centre for NMR Spectroscopy of Biological Macromolecules at Carlsberg Laboratory. Spectra were acquired using standard pulse sequences. The deuterated solvent was DMSO-*d*_*6*_ and signals were referenced by solvent signals for DMSO-*d*_*6*_ at δ_H_ = 2.50 ppm and δ_C_ = 39.5 ppm. The NMR data was processed in MestReNova V.10.0.2–15465. Chemical shifts are reported in ppm (*δ*) and scalar couplings are reported in hertz (Hz). The sizes of the *J* coupling constants in the tables are the experimentally measured values from the 1D ^1^H and DQF-COSY spectra. There are minor variations in the measurements, which may be explained by the uncertainty of *J* and the spectral digital resolution. Descriptions of NMR structural elucidations of niduclavin and niduporthin are provided in S2 Text and NMR data are provided in S1 Dataset.

## Supporting Information

S1 Dataset Tandem MS and NMR structural elucidation data. (XLSX)

S1 Fig Proposed mechanism for [4+2]-cycloaddition of niduclavin. A) Formation of the cytochalasin core structure through a [4+2]-cycloadition as proposed by Qiao et al. [1]. B) Proposed mechanism for the [4+2]-cycloaddition-mediated formation of niduclavin.
(DOCX)

S2 Fig Southern blot for strains carrying various PKS-NRPS variants. A) The probe hybridizes to the *pyrG* marker and the downstream region. A band of 3.1 kb indicates correct integration of the gene. B) Southern blot of PKS-NRPS variants. Lane1: NID3 reference strain carrying a copy of *pyrG* in the *nkuA* locus, lane 2: *ccsA* WT, lane 3: *syn2* WT, lane 4–9: *ccsA-syn2* chimeric genes, lane 10: *syn2-ccsA* chimeric gene, lane 11–16: various adenylation domain point mutations of ccsA. Unspecific binding (UB) of the probe is observable for samples of high DNA concentration. Since extra copies would be integrated ectopically it is unlikely that two extra bands of identical size for several independent transformants would be seen. Additionally, in the case of extra copies, the intensities of the bands are expected to be equal. Therefore, it was concluded that the bands seen for samples with high DNA concentration represent unspecific binding of the probe. As shown, the *syn2-(GC)* strain carries two copies of the transformed gene. See also Supplemental Experimental Procedures.
(DOCX)

S3 Fig Base peak chromatograms of A. nidulans extracts expressing various linker modified variants. Base peak chromatograms of A) *ccsA* and B) *syn2*. Highlighted areas represent EICs for A) niduclavin (*m/z* 416.2584), and B) niduporthin (*m/z* 427.2380).
(DOCX)

S4 Fig Fluorescence tagging of individually expressed CcsA PKS- and NRPS modules. The RFP-tagged PKS module and the mCitrine-tagged NRPS module both appear to be localized to the cytoplasm. C-terminal RFP-tagging of the PKS module and C-terminal mCitrine-tagging of the NRPS module indicate cytoplasmic localization for both modules. N-terminal tagging of the modules revealed the same localization as seen for the C-terminal tagging (results not shown). Scale bar 10 µm. See also Supplemental Experimental Procedures.
(DOCX)

S5 Fig Constructed fusions between CcsA and Syn2 PKS- and NRPS modules. A) Schematic illustration of the fusions between CccA and Syn2 PKS- and NRPS modules. Arrows indicate the point of fusion. B) Base peak chromatograms of A. nidulans extracts expressing CcsA-Syn2 chimeric PKS-NRPSs. Niduchimaeralin A elutes as several isomeric structures and are highlighted in blue (EIC @ m/z 455.2693).
(DOCX)

S1 Protocol Southern blot. (DOCX)

S2 Protocol Microscopy. (DOCX)

S1 Table List of fungal strains. (DOCX)

S2 Table List of primers. (DOCX)

S1 Text Fragmentation patterns of niduclavin, niduporthin, and niduchimaeralin A and B. (DOCX)

S2 Text NMR structural elucidations of niduclavin and niduporthin. (DOCX)
