# Supplementary material for: Linker Flexibility Facilitates Module Exchange in Fungal Hybrid PKS-NRPS Engineering
Source: PLoS One. 2016 Aug 23;11(8):e0161199. doi: 10.1371/journal.pone.0161199 (PMC4994942; doi:10.1371/journal.pone.0161199)
Supplement: S1 Fig — A) Formation of the cytochalasin core structure through a [4+2]-cycloadition as proposed by Qiao et al. [1]. B) Proposed mechanism for the [4+2]-cycloaddition-mediated formation of niduclavin. (DOCX) [file pone.0161199.s002.docx]

**S1 Fig. Proposed mechanism for [4+2]-cycloaddition of niduclavin.** A) Formation of the cytochalasin core structure through a [4+2]-cycloadition as proposed by Qiao et al. [1]. B) Proposed mechanism for the [4+2]-cycloaddition-mediated formation of niduclavin.

A)

B)

1. Qiao K, Tang Y, Chooi Y-H. Identification and engineering of the cytochalasin gene cluster from Aspergillus clavatus NRRL 1. Metab Eng. Elsevier; 2011;13: 723–732. doi:10.1016/j.ymben.2011.09.008
