# Supplementary material for: Linker Flexibility Facilitates Module Exchange in Fungal Hybrid PKS-NRPS Engineering
Source: PLoS One. 2016 Aug 23;11(8):e0161199. doi: 10.1371/journal.pone.0161199 (PMC4994942; doi:10.1371/journal.pone.0161199)

**S2 Fig. Southern blot for strains carrying various PKS-NRPS variants.** A) The probe hybridizes to the pyrG marker and the downstream region. A band of 3.1 kb indicates correct integration of the gene. B) Southern blot of PKS-NRPS variants. Lane1: NID3 reference strain carrying a copy of pyrG in the nkuA locus, lane 2: ccsA WT, lane 3: syn2 WT, lane 4-9: ccsA-syn2 chimeric genes, lane 10: syn2-ccsA chimeric gene, lane 11-16: various adenylation domain point mutations of ccsA. Unspecific binding (UB) of the probe is observable for samples of high DNA concentration. Since extra copies would be integrated ectopically it is unlikely that two extra bands of identical size for several independent transformants would be seen. Additionally, in the case of extra copies, the intensities of the bands are expected to be equal. Therefore, it was concluded that the bands seen for samples with high DNA concentration represent unspecific binding of the probe. As shown, the syn2-(GC) strain carries two copies of the transformed gene. See also Supplemental Experimental Procedures.

A)


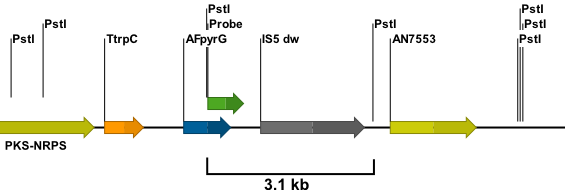


B)


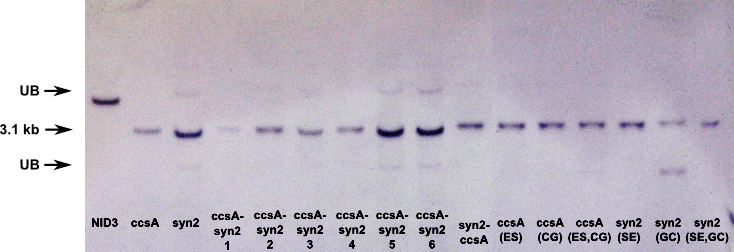

Supplement: S2 Fig — A) The probe hybridizes to the pyrG marker and the downstream region. A band of 3.1 kb indicates correct integration of the gene. B) Southern blot of PKS-NRPS variants. Lane1: NID3 reference strain carrying a copy of pyrG in the nkuA locus, lane 2: ccsA WT, lane 3: syn2 WT, lane 4–9: ccsA-syn2 chimeric genes, lane 10: syn2-ccsA chimeric gene, lane 11–16: various adenylation domain point mutations of ccsA. Unspecific binding (UB) of the probe is observable for samples of high DNA concentration. Since extra copies would be integrated ectopically it is unlikely that two extra bands of identical size for several independent transformants would be seen. Additionally, in the case of extra copies, the intensities of the bands are expected to be equal. Therefore, it was concluded that the bands seen for samples with high DNA concentration represent unspecific binding of the probe. As shown, the syn2-(GC) strain carries two copies of the transformed gene. See also Supplemental Experimental Procedures. (DOCX) [file pone.0161199.s003.docx]
