# Supplementary material for: Linker Flexibility Facilitates Module Exchange in Fungal Hybrid PKS-NRPS Engineering
Source: PLoS One. 2016 Aug 23;11(8):e0161199. doi: 10.1371/journal.pone.0161199 (PMC4994942; doi:10.1371/journal.pone.0161199)
Supplement: S3 Fig — Base peak chromatograms of A) ccsA and B) syn2. Highlighted areas represent EICs for A) niduclavin (m/z 416.2584), and B) niduporthin (m/z 427.2380). (DOCX) [file pone.0161199.s004.docx]

**S3 Fig. Base peak chromatograms of A. nidulans extracts expressing various linker modified variants.** Base peak chromatograms of A) ccsA and B) syn2. Highlighted areas represent EICs for A) niduclavin (m/z 416.2584), and B) niduporthin (m/z 427.2380).

reference

*ccsA* (WT)

*ccsA*-CAC

*ccsA*-CEC

*ccsA*-CMC

*ccsA*-LΔ150

*ccsA*-LΔ225up

*ccsA*-LΔ225dw

*ccsA*-L-GSG

*ccsA*-RFPlink1

*ccsA*-RFPlink2

8.6

8.8

9.0

9.2

9.4

9.6

[min]

A)

reference

*syn2* (WT)

*syn2*-L-GSG

7.50

8.00

8.50

9.00

[min]

B)
