# Supplementary material for: Linker Flexibility Facilitates Module Exchange in Fungal Hybrid PKS-NRPS Engineering
Source: PLoS One. 2016 Aug 23;11(8):e0161199. doi: 10.1371/journal.pone.0161199 (PMC4994942; doi:10.1371/journal.pone.0161199)
Supplement: S4 Fig — The RFP-tagged PKS module and the mCitrine-tagged NRPS module both appear to be localized to the cytoplasm. C-terminal RFP-tagging of the PKS module and C-terminal mCitrine-tagging of the NRPS module indicate cytoplasmic localization for both modules. N-terminal tagging of the modules revealed the same localization as seen for the C-terminal tagging (results not shown). Scale bar 10 µm. See also Supplemental Experimental Procedures. (DOCX) [file pone.0161199.s005.docx]

**S4 Fig.** **Fluorescence tagging of individually expressed CcsA PKS- and NRPS modules**. The RFP-tagged PKS module and the mCitrine-tagged NRPS module both appear to be localized to the cytoplasm. C-terminal RFP-tagging of the PKS module and C-terminal mCitrine-tagging of the NRPS module indicate cytoplasmic localization for both modules. N-terminal tagging of the modules revealed the same localization as seen for the C-terminal tagging (results not shown). Scale bar 10 µm. See also Supplemental Experimental Procedures.


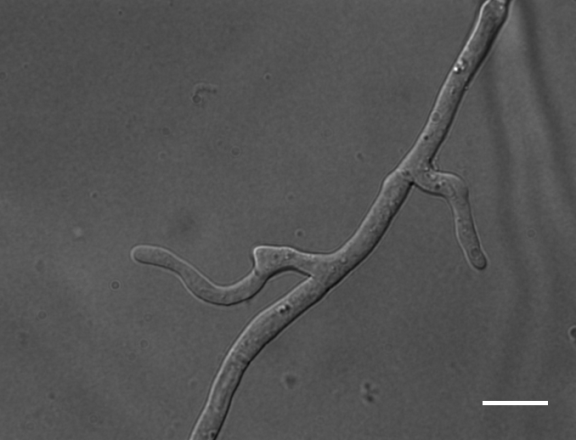

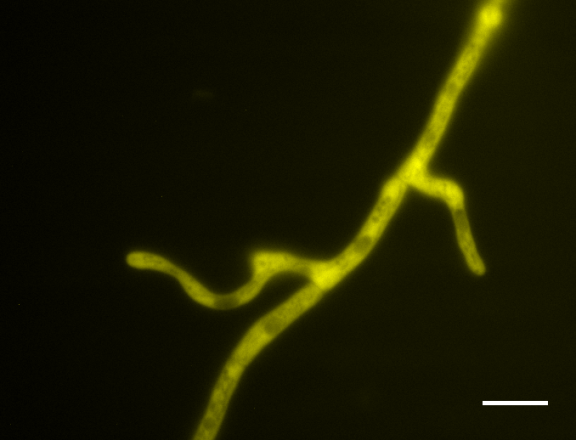


**Fluorescence**

**DIC**


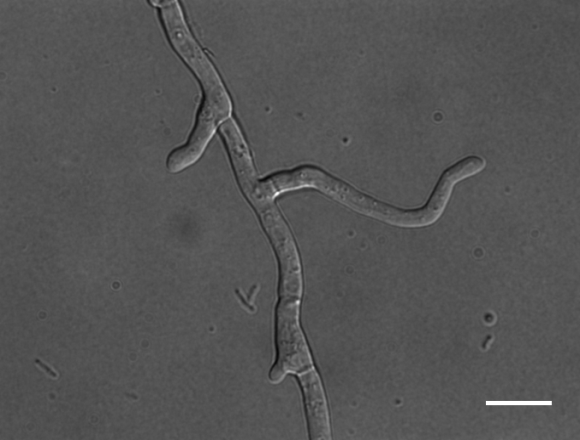

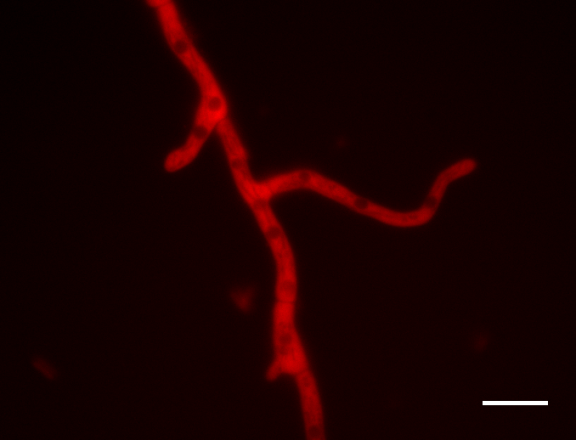


CcsA(PKS)-RFP

CcsA(NRPS)-

mCitrine
