# Supplementary material for: Linker Flexibility Facilitates Module Exchange in Fungal Hybrid PKS-NRPS Engineering
Source: PLoS One. 2016 Aug 23;11(8):e0161199. doi: 10.1371/journal.pone.0161199 (PMC4994942; doi:10.1371/journal.pone.0161199)
Supplement: S5 Fig — A) Schematic illustration of the fusions between CccA and Syn2 PKS- and NRPS modules. Arrows indicate the point of fusion. B) Base peak chromatograms of A. nidulans extracts expressing CcsA-Syn2 chimeric PKS-NRPSs. Niduchimaeralin A elutes as several isomeric structures and are highlighted in blue (EIC @ m/z 455.2693). (DOCX) [file pone.0161199.s006.docx]

**S5 Fig.** **Constructed fusions between CcsA and Syn2 PKS- and NRPS modules.** A) Schematic illustration of the fusions between CccA and Syn2 PKS- and NRPS modules. Arrows indicate the point of fusion. B) Base peak chromatograms of A. nidulans extracts expressing CcsA-Syn2 chimeric PKS-NRPSs. Niduchimaeralin A elutes as several isomeric structures and are highlighted in blue (EIC @ m/z 455.2693).


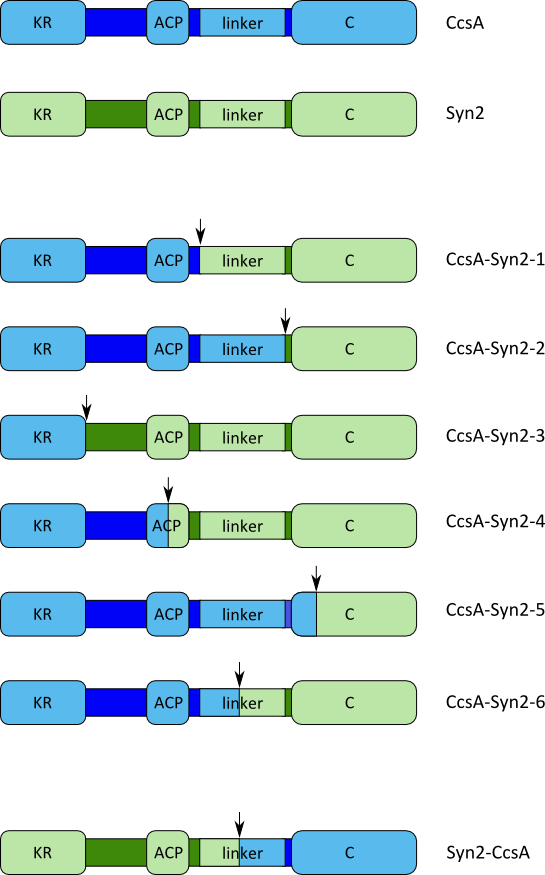


A)

B)

NID3

CM1

CM2

CM3

CM4

CM5

CM6

8.0

8.5

9.0

9.5

10.0

10.5

11.0

[min]
