# Supplementary material for: Linker Flexibility Facilitates Module Exchange in Fungal Hybrid PKS-NRPS Engineering
Source: PLoS One. 2016 Aug 23;11(8):e0161199. doi: 10.1371/journal.pone.0161199 (PMC4994942; doi:10.1371/journal.pone.0161199)
Supplement: S1 Protocol — (DOCX) [file pone.0161199.s007.docx]

**S1 Protocol. Southern blot**

DNA for southern blot was extracted as follows: Spores were harvested from plates of MM supplemented with arginine and grown for 2 days in shake flasks at 37°C. The biomass was filtered and freeze-dried overnight, and the freeze-dried mycelium was grinded in a mortar. For each strain, 10 ml lysis buffer was added consisting of 3.75 ml of buffer A (0.35 M sorbitol, 0.1 M Tris-HCl pH 9, 5 mM EDTA pH 8), 3.75 ml of pre-heated (65°C) buffer B (0.2 M Tris-HCl pH 9, 50 mM EDTA pH 8, 2 M NaCl, 2 % CTAB), 1.5 ml 5 % Sarkosyl, 1 ml 1 % PVP and 100 µl Proteinase K. The samples were vortexed and incubated at 65°C for 30 minutes followed by the addition of 3.35 ml of 5 M potassium acetate, and subsequent incubation on ice for 30 minutes. The lysates were centrifuged for 30 minutes at 5000 g at 4°C and 5 ml of phenol:chloroform:iso-amylalchohol (25:24:1) was added to the supernatant. The samples were centrifuged for 20 minutes at 4°C and the aqueous phase was transferred to new tubes and 1/10 volume of 3 M sodium acetate, and 1 volume of isopropanol was added followed by centrifugation for 30 minutes at 4°C. The pellet was washed with 2 ml 70 % ethanol and finally re-dissolved in 600 µl TE buffer.

For each southern blot sample, 2 µg of DNA was digested with *Pst*I, and the blot was performed as described by Sambrook and Russell [1]. For generation of the probe, a 692 bp DNA fragment was PCR amplified using the primers AFpyrG-F and AFpyrG-R and the Biotin DecaLabel DNA Labelling Kit (Thermo Scientific) was used for incorporation of biotin. Detection was achieved using the Biotin Chromogenic detection kit (ThermoFisher Scientific) using streptavidin conjugated to alkaline phosphatase.

1. Sambrook, J., Russell DW. Molecular cloning a laboratory manual. Cold Spring Habor, NY.: Cold Spring Harbor Laboratory Press; 2001.
