# Supplementary material for: Linker Flexibility Facilitates Module Exchange in Fungal Hybrid PKS-NRPS Engineering
Source: PLoS One. 2016 Aug 23;11(8):e0161199. doi: 10.1371/journal.pone.0161199 (PMC4994942; doi:10.1371/journal.pone.0161199)
Supplement: S2 Protocol — (DOCX) [file pone.0161199.s008.docx]

**S2 Protocol. Microscopy**

MM agar slides were prepared by covering with 1 ml MM agar (with necessary supplements), then inoculated with spores and incubated at 30°C in petri dishes overnight. A cooled Evolution QEi monochrome digital camera (Media Cybernetics Inc.) mounted on a Nikon Eclipse E1000 microscope (Nikon) captured live-cell images using a Plan-Fluor x100, 1.30 numerical aperture objective lens. The illumination source was a 103-watt mercury arc lamp (Osram). The fluorophores RFP and mCitrine were visualized using a band pass RFP (EX545/30, EM620/60; Nikon) and YFP filter (EX500/20, EM535/30; Nikon), respectively. Exposure time for images was 500 msec. Red and yellow colors were added to the corresponding fluorescence signals using image processing in ImageJ.
