# Supplementary material for: Linker Flexibility Facilitates Module Exchange in Fungal Hybrid PKS-NRPS Engineering
Source: PLoS One. 2016 Aug 23;11(8):e0161199. doi: 10.1371/journal.pone.0161199 (PMC4994942; doi:10.1371/journal.pone.0161199)
Supplement: S1 Table — (DOCX) [file pone.0161199.s009.docx]

# S1 Table. List of fungal strains

| # | Strain identifier | Genotype |  |
| --- | --- | --- | --- |
| 1 | nkuAΔ | *argB2, pyrG89, veA1, nkuA∆* |  |
| 2 | nkuA-trS | *argB2, pyrG89, veA1, nkuA-trS::pyrG* |  |
| 3 | ccsA prepop | *argB2, pyrG89, veA1, nkuA∆, IS1::PgpdA-ccsA-TtrpC::AFpyrG* |  |
| 4 | syn2 prepop | *argB2, pyrG89, veA1, nkuA∆, IS5::PgpdA-syn2-TtrpC::AFpyrG* |  |
| 5 | ccsC prepop | *argB2, pyrG89, veA1, nkuA∆, IS2::PgpdA-ccsC-TtrpC::AFpyrG* |  |
| 6 | ccsC pop | *argB2, pyrG89, veA1, nkuA∆, IS2::PgpdA-ccsC-TtrpC* |  |
| 7 | rap2 prepop | *argB2, pyrG89, veA1, nkuA∆, IS2::PgpdA-rap2-TtrpC::AFpyrG* |  |
| 8 | rap2 pop | *argB2, pyrG89, veA1, nkuA∆, IS2::PgpdA-rap2-TtrpC* |  |
| 9 | ccsA, ccsC prepop | *argB2, pyrG89, veA1, nkuA∆, IS5::PgpdA-ccsA-TtrpC::AFpyrG, IS2::PgpdA-ccsC-TtrpC* |  |
| 10 | syn2, rap2 prepop | *argB2, pyrG89, veA1, nkuA∆, IS5::PgpdA-syn2-TtrpC::AFpyrG, IS2::PgpdA-rap2-TtrpC* |  |
| 11 | ccsA-syn2-1, ccsC prepop | *argB2, pyrG89, veA1, nkuA∆, IS5::PgpdA-CM1-TtrpC::AFpyrG, IS2::PgpdA-ccsC-TtrpC* |  |
| 12 | ccsA-syn2-2, ccsC prepop | *argB2, pyrG89, veA1, nkuA∆, IS5::PgpdA-CM2-TtrpC::AFpyrG, IS2::PgpdA-ccsC-TtrpC* |  |
| 13 | ccsA-syn2-3, ccsC prepop | *argB2, pyrG89, veA1, nkuA∆, IS5::PgpdA-CM3-TtrpC::AFpyrG, IS2::PgpdA-ccsC-TtrpC* |  |
| 14 | ccsA-syn2-4, ccsC prepop | *argB2, pyrG89, veA1, nkuA∆, IS5::PgpdA-CM4-TtrpC::AFpyrG, IS2::PgpdA-ccsC-TtrpC* |  |
| 15 | ccsA-syn2-5, ccsC prepop | *argB2, pyrG89, veA1, nkuA∆, IS5::PgpdA-CM5-TtrpC::AFpyrG, IS2::PgpdA-ccsC-TtrpC* |  |
| 16 | ccsA-syn2-6, ccsC prepop | *argB2, pyrG89, veA1, nkuA∆, IS5::PgpdA-CM6-TtrpC::AFpyrG, IS2::PgpdA-ccsC-TtrpC* |  |
| 17 | syn2-ccsA, rap2 prepop | *argB2, pyrG89, veA1, nkuA∆, IS5::PgpdA-MC6-TtrpC::AFpyrG, IS2::PgpdA-rap2-TtrpC* |  |
| 18 | ΔasqJ, ccsA, ccsC prepop | *argB2, pyrG89, veA1, nkuA∆, AN9227∆::AFpyrG, IS1::PgpdA-ccsA-TtrpC, IS2::PgpdA-ccsC-TtrpC* |  |
| 19 | CAC, ccsC prepop | *argB2, pyrG89, veA1, nkuA∆, IS5::PgpdA-CAC-TtrpC::AFpyrG, IS2::PgpdA-ccsC-TtrpC* |  |
| 20 | CEC, ccsC prepop | *argB2, pyrG89, veA1, nkuA∆, IS5::PgpdA-CEC-TtrpC::AFpyrG, IS2::PgpdA-ccsC-TtrpC* |  |
| 21 | CMC, ccsC prepop | *argB2, pyrG89, veA1, nkuA∆, IS5::PgpdA-CMC-TtrpC::AFpyrG, IS2::PgpdA-ccsC-TtrpC* |  |
| 22 | ccsA(LΔ150), ccsC prepop | *argB2, pyrG89, veA1, nkuA∆, IS5::PgpdA-ccsA(LD150)-TtrpC::AFpyrG, IS2::PgpdA-ccsC-TtrpC* |  |
| 23 | ccsA(LΔ225up), ccsC prepop | *argB2, pyrG89, veA1, nkuA∆, IS5::PgpdA-ccsA(LD225up)-TtrpC::AFpyrG, IS2::PgpdA-ccsC-TtrpC* |  |
| 24 | ccsA(LΔ225dw), ccsC prepop | *argB2, pyrG89, veA1, nkuA∆, IS5::PgpdA-ccsA(LD225dw)-TtrpC::AFpyrG, IS2::PgpdA-ccsC-TtrpC* |  |
| 25 | ccsA(L-GSG), ccsC prepop | *argB2, pyrG89, veA1, nkuA∆, IS5::PgpdA-ccsA(L-GSG)-TtrpC::AFpyrG, IS2::PgpdA-ccsC-TtrpC* |  |
| 26 | syn2(L-GSG), rap2 prepop | *argB2, pyrG89, veA1, nkuA∆, IS5::PgpdA-syn2(L-GSG)-TtrpC::AFpyrG, IS2::PgpdA-rap2-TtrpC* |  |
| 27 | ccsA-RFPlink1, ccsC prepop | *argB2, pyrG89, veA1, nkuA∆, IS5::PgpdA-ccsA-RFPlink1-TtrpC::AFpyrG, IS2::PgpdA-ccsC-TtrpC* |  |
| 28 | ccsA-RFPlink2, ccsC prepop | *argB2, pyrG89, veA1, nkuA∆, IS5::PgpdA-ccsA-RFPlink2-TtrpC::AFpyrG, IS2::PgpdA-ccsC-TtrpC* |  |
| 29 | ccsA(PKS)-RFP prepop | *argB2, pyrG89, veA1, nkuA∆, IS5::PgpdA-ccsA(PKS)-RFP-TtrpC::AFpyrG* |  |
| 30 | ccsA(NRPS)-mCitrine prepop | *argB2, pyrG89, veA1, nkuA∆, IS2::PgpdA-ccsA(NRPS)-mCitrine-TtrpC::AFpyrG* |  |

| # | Strain notes |
| --- | --- |
| 1 | Permanent *nku* deletion strain (background strain) |
| 2 | Transient small repeat in *nkuA* (reference strain for chemical analysis) |
| 3 | Oex of *A. clavatus* *ccsA* (ACLA_078660) from the *ccs* gene cluster |
| 4 | Oex of *M. oryzae* PKS-NRPS *syn2* (CAG_28798) |
| 5 | Oex of *A. clavatus* enoyl reductase *ccsC* (ACLA_078700) from the *ccs* gene cluster |
| 6 | Oex of *A. clavatus* enoyl reductase *ccsC* (ACLA_078700) from the *ccs* gene cluster |
| 7 | Oex of *M. oryzae* enoyl reductase *rap2* (MGG_08380) |
| 8 | Oex of *M. oryzae* enoyl reductase *rap2* (MGG_08380) |
| 9 | Oex of *A. clavatus* *ccsA* (ACLA_078660) and *ccsC* (ACLA_078700) |
| 10 | Oex of *M. oryzae* *syn2* (CAG_28798) and *rap2* (MGG_08380) |
| 11 | Oex of chimeric PKS-NRPS, *ccsA* PKS fused with *syn2* NRPS moiety |
| 12 | Oex of chimeric PKS-NRPS, *ccsA* PKS fused with *syn2* NRPS moiety |
| 13 | Oex of chimeric PKS-NRPS, *ccsA* PKS fused with *syn2* NRPS moiety |
| 14 | Oex of chimeric PKS-NRPS, *ccsA* PKS fused with *syn2* NRPS moiety |
| 15 | Oex of chimeric PKS-NRPS, *ccsA* PKS fused with *syn2* NRPS moiety |
| 16 | Oex of chimeric PKS-NRPS, *ccsA* PKS fused with *syn2* NRPS moiety |
| 17 | Oex of chimeric PKS-NRPS, *syn2* PKS fused with *ccsA* NRPS moiety |
| 18 | Deletion of AN9227 in *ccsA, ccsC* background strain |
| 19 | Oex of *ccsA* with linker swapped for *A. nidulans* *apdA* (AN8412), co-expressed with enoyl reductase *ccsC* |
| 20 | Oex of *ccsA* with linker swapped for *A. clavatus eqiS* (ACLA_023380) linker, co-expressed with enoyl reductase *ccsC* |
| 21 | Oex of *ccsA* with linker swapped for *M. oryzae* *syn2*, co-expressed with enoyl reductase *ccsC* |
| 22 | Oex of *ccsA* with truncated linker, co-expressed with enoyl reductase *ccsC* |
| 23 | Oex of *ccsA* with truncated linker, co-expressed with enoyl reductase *ccsC* |
| 24 | Oex of *ccsA* with truncated linker, co-expressed with enoyl reductase *ccsC* |
| 25 | Oex of *ccsA* without linker (only GSG linker), co-expressed with enoyl reductase *ccsC* |
| 26 | Oex of *syn2* without linker (only GSG linker), co-expressed with enoyl reductase *rap2* |
| 27 | Oex of *A. clavatus* *ccsA* with RFP in the linker, co-expressed with enoyl reductase *ccsC* |
| 28 | Oex of *A. clavatus ccsA* with RFP as the linker, co-expressed with enoyl reductase *ccsC* |
| 29 | Oex of RFP-tagged PKS module from *ccsA* |
| 30 | Oex of mCitrine-tagged NRPS module from *ccsA* |
