# Supplementary material for: Linker Flexibility Facilitates Module Exchange in Fungal Hybrid PKS-NRPS Engineering
Source: PLoS One. 2016 Aug 23;11(8):e0161199. doi: 10.1371/journal.pone.0161199 (PMC4994942; doi:10.1371/journal.pone.0161199)
Supplement: S2 Table — (DOCX) [file pone.0161199.s010.docx]

**S2 Table. List of Primers**

| **Name** | **Sequence** | **Purpose** |  |  |  |
| --- | --- | --- | --- | --- | --- |
| ACLAccsA-eOex-1FU_2 | GGGTTTAAUATGGGGTCATTTCAGAACTCCTC | ccsA into pU2115-5 | |  |  |
| ACLAccsA-eOex-2FU | AGAGAGGAAUGGACTGGAATGGCATCACAAAC | ccsA into pU2115-5 | |  |  |
| ACLAccsA-eOex-2RU | ATTCCTCTCUCTCTCGGGAACCGCTTCAC | ccsA into pU2115-5 | |  |  |
| ACLAccsA-eOex-3FU | AAGGATTUGGCCGCAGAGGAGAAGCA | ccsA into pU2115-5 | |  |  |
| ACLAccsA-eOex-3RU | AAATCCTUAGCCACAACAATGTTCCGTGA | ccsA into pU2115-5 | |  |  |
| ccsA-Pac-RU | GGTCTTAAUTGCTGTGTCCCAATCAGACGT | ccsA into pU2115-5 | |  |  |
| ACLA-ccsC-FU | AGAGCGAUATGACCGTACCAACCACTATCCG | ccsC into pU2111-2 (p60) | | |  |
| ACLA-ccsC-RU | TCTGCGAUTTACATGCCGATGCTCACAACC | ccsC into pU2111-2 (p60) | | |  |
| SYN2-Pac-FU | GGGTTTAAUATGGAAGCCCAGAATGAGCC | syn2 into pU2115-5 | |  |  |
| SYN2-RU | ACCGAGACTCUTGCCAGAGCGTTCGGCT | syn2 into pU2115-5 | |  |  |
| SYN2-2FU | AGAGTCTCGGUTGTCAAAAGCCTTCAGGACGA | syn2 into pU2115-5 | |  |  |
| SYN2-2RU | AAACAAAAGUCCGAACCTCATCTGCTCGTT | syn2 into pU2115-5 | |  |  |
| SYN2-3FU | ACTTTTGTTUGGCGGTCTTCCTGGAGC | syn2 into pU2115-5 | |  |  |
| SYN2-3RU | ATGAGTTCCUTGGGCAGCAGCTTCTGAG | syn2 into pU2115-5 | |  |  |
| SYN2-4FU | AGGAACTCAUACCCAAATTCGATGAGAAGG | syn2 into pU2115-5 | |  |  |
| SYN2-4RU | ACCAAAGUGGCGGGCCATTGTGG | syn2 into pU2115-5 | |  |  |
| SYN2-5FU | ACTTTGGUGCATCGCATCGACGACAT | syn2 into pU2115-5 | |  |  |
| SYN2-Pac-5RU | GGTCTTAAUTCAATGCCAAGCCTCTCCC | syn2 into pU2115-5 | |  |  |
| RAP2-Pac-FU | GGGTTTAAUATGTATATTCCTTCGGCGAGGA | rap2 into pU2115-2 | |  |  |
| RAP2-Pac-RU | GGTCTTAAUCTATTCAAGTGATACAACCAGCTTCTG | rap2 into pU2115-2 | |  |  |
| ACLAccsA-eOex-1FU_2 | GGGTTTAAUATGGGGTCATTTCAGAACTCCTC | CM chimeras into pU2115-5 | | |  |
| ACLAccsA-eOex-2FU | AGAGAGGAAUGGACTGGAATGGCATCACAAAC | CM chimeras into pU2115-5 | | |  |
| ACLAccsA-eOex-2RU | ATTCCTCTCUCTCTCGGGAACCGCTTCAC | CM chimeras into pU2115-5 | | |  |
| SYN2-Pac-5RU | GGTCTTAAUTCAATGCCAAGCCTCTCCC | CM chimeras into pU2115-5 | | |  |
| CM1-mid-RU | AGCGTTGGGAUCCAGGTTCGGG | CM chimeras into pU2115-5 | | |  |
| CM1-end-FU | ATCCCAACGCUACTGGCCTCAGCAAGC | CM chimeras into pU2115-5 | | |  |
| CM2-mid-RU | ACCGGTTTGGUCTTTGTAATGATTTGGCTGGCA | CM chimeras into pU2115-5 | | |  |
| CM2-end-FU | ACCAAACCGGUGGAGATCACCAAGAGCGGG | CM chimeras into pU2115-5 | | |  |
| CM3-mid-RU | ATGGCACCAAUATGCACAGC | CM chimeras into pU2115-5 | | |  |
| CM3-end-FU | ATTGGTGCCAUCTTTGGCAACGGCTACGTCA | CM chimeras into pU2115-5 | | |  |
| CM4-mid-RU | ACCAGCGAGUCAATTCCCAA | CM chimeras into pU2115-5 | | |  |
| CM4-end-FU | ACTCGCTGGUGGCCGTTGACATCCGATCCT | CM chimeras into pU2115-5 | | |  |
| CM5-mid-RU | AGGGCCTCGUGACGCTGTCCGACGACTTGAA | CM chimeras into pU2115-5 | | |  |
| CM5-end-FU | ACGAGGCCCUCCGCACC | CM chimeras into pU2115-5 | | |  |
| CM6-mid-RU | ATCTTGCTAUCCTTTGGTGCAGCCT | CM chimeras into pU2115-5 | | |  |
| CM6-end-FU | ATAGCAAGAUCTCCAGCCCCAAATCC | CM chimeras into pU2115-5 | | |  |
| CM7-mid-RU | AGCTTCGGGGUCAACTCCTTGGG | CM chimeras into pU2115-5 | | |  |
| CM7-end-FU | ACCCCGAAGCUGCAGGAGGCTTTGC | CM chimeras into pU2115-5 | | |  |
| SYN2-Pac-FU | GGGTTTAAUATGGAAGCCCAGAATGAGCC | MC6 chimera into pU2115-5 | | |  |
| SYN2-2RU | AAACAAAAGUCCGAACCTCATCTGCTCGTT | MC6 chimera into pU2115-5 | | |  |
| SYN2-3FU | ACTTTTGTTUGGCGGTCTTCCTGGAGC | MC6 chimera into pU2115-5 | | |  |
| ccsA-Pac-RU | GGTCTTAAUTGCTGTGTCCCAATCAGACGT | MC6 chimera into pU2115-5 | | |  |
| SYN2-6RU | ATATTGGTCAUCAGCTGGTATCAACACTGAC | MC6 chimera into pU2115-5 | | |  |
| ccsA-10FU | ATGACCAATAUCCAACAGCACTTGAGACTC | MC6 chimera into pU2115-5 | | |  |
| ccsA-7RU | ATTGGGAUCCAGGTTCGGG | Linker swap/modification in ccsA | | | |
| ccsA_NRPS-2FU | AAGAAGAGTGUTCCTATGGCGTTT | Linker swap/modification in ccsA | | | |
| SYN2_linker-FU | ATCCCAAUGCTACTGGCCTCAGCAAGC | Linker swap/modification in ccsA | | | |
| SYN2_linker-RU | ACACTCTTCTUGACCTCGAAGCCGGCGCTGGC | Linker swap/modification in ccsA | | | |
| EqiS_linker-FU | ATCCCAAUGCGCTAACAAACACCAAGATTG | Linker swap/modification in ccsA | | | |
| EqiS_linker-RU | ACACTCTTCTUGACCTCCTTAGCTGCCTGTGAGACTTTAATATT | Linker swap/modification in ccsA | | | |
| ApdA_linker-FU | ATCCCAAUCAGCTGGAGAAGCAAGACACG | Linker swap/modification in ccsA | | | |
| ApdA_linker-RU | ACACTCTTCTUGACCTCGGAGGCAGGCTCAGCG | Linker swap/modification in ccsA | | | |
| CNC_linker-FU | ATCCCAAUTCCCCTGCGACAGTGTCG | Linker swap/modification in ccsA | | | |
| CNC_linker-RU | ACACTCTTCTUGACCTCCGCGGGAGAATCGGGTT | Linker swap/modification in ccsA | | | |
| ccsA-8RU | ATGCTTCTCAUTGGTCTTGATCATGTTCTGCAC | Linker swap/modification in ccsA | | | |
| ccsA-3FU | ATGAGAAGCAUCTCACCGATCAGGAACCCG | Linker swap/modification in ccsA | | | |
| ccsA-9RU | AAGTGCTGUGTTGGGATCCAGGTTCGGG | Linker swap/modification in ccsA | | | |
| ccsA-4FU | ACAGCACTUGAGACTCCATCAAAG | Linker swap/modification in ccsA | | | |
| ccsA-10RU | ACCTCTGGAUATTGGCTATCCTTTGGTG | Linker swap/modification in ccsA | | | |
| ccsA-5FU | ATCCAGAGGUCAAGAAGAGTGTTCCTATGG | Linker swap/modification in ccsA | | | |
| ccsA-11RU | ACCTCGCCUGAGCCGTTGGGATCCAGGTTCGGG | Linker swap/modification in ccsA | | | |
| ccsA-6FU | AGGCGAGGUCAAGAAGAGTGTTCCTATGG | Linker swap/modification in ccsA | | | |
| asqJup-FU | GGGTTTAAUGTTTTGGTAGAGAAGAATGGATGG | asqJ deletion in A. nidulans | | |  |
| asqJup-RU | GGACTTAAUTGTTGCCTGAGAAGATGGGC | asqJ deletion in A. nidulans | | |  |
| asqJdw-FU | GGCATTAAUTTCTTTAGAGATCTTCCTCCTAATACAG | asqJ deletion in A. nidulans | | |  |
| asqJdw-RU | GGTCTTAAUCGATAGATATTGTCGTTGATGGG | asqJ deletion in A. nidulans | | |  |
| AFpyrG-F | GGCATCGTCGAGGCTCTG | Construction of probe for southern blot | | | |
| AFpyrG-R | GCTCGGTCGTTCGGCTG | Construction of probe for southern blot | | | |

Primers marked in blue are repeated.
