# Supplementary material for: Linker Flexibility Facilitates Module Exchange in Fungal Hybrid PKS-NRPS Engineering
Source: PLoS One. 2016 Aug 23;11(8):e0161199. doi: 10.1371/journal.pone.0161199 (PMC4994942; doi:10.1371/journal.pone.0161199)
Supplement: S1 Text — (DOCX) [file pone.0161199.s011.docx]

# S1 Text. Fragmentation patterns of niduclavin, niduporthin, and niduchimaeralin A and B.

Based on high resolution tandem MS experiments it was possible to give tentative structures for the two hybrid products niduchimaeralin A and B, when compared to those for niduclavin and niduporthin. MS^2^ was done on all four products to help verify the fragmentation patterns observed. In addition, MS^3^ experiments were performed on the ions *m/z* 175 and *m/z* 203, to further verify the fragmentation patterns of these ions (data not shown).

As niduclavin and niduporthin are proposed to be formed via [4+2] cycloadditions, retro-Diels Alder derived fragments were expected. This, however, was not the case. Rather it would seem that a McLafferty rearrangement provided the basis for most fragments as have also been showing for the structurally related chaetoglobosins (Xu et al., 2012).

Both niduclavin and niduporthin show fragments in line with an initial McLafferty rearrangement, followed by a variety of fragmentation pathways. The most predominant fragments for **niduclavin** are water loss, and water loss followed by loss of a butenyl group, both of which would be possible with or without a preceding McLafferty rearrangement. Furthermore, an ion with a mass-to-charge ratio of 203 is expected to correspond to part of the polyketide after a McLafferty rearrangement, with additional fragments matching consecutive CH_2_ losses (*m/z* = 189, 175, 161, 147, 133, and 119), also confirmed by MS^3^-experiments.

The major fragments in the **niduporthin** spectra are water loss, and a peak corresponding to water loss followed by propene loss, similar to what was observed for niduclavin. Moreover, a fragment with an m/z of 175 matches fragmentation following a McLafferty rearrangement, followed by ion matching succeeding CH_2_ losses (*m/z* = 147 (2x CH_2_), 133, 119, 105), also confirmed by MS^3^-experiments.

**Niduchimaeralin A** was expected to share PK backbone with niduclavin, and differ by the incorporation of tryptophan instead of phenylalanine. Both compounds share a fragment ion with an *m/z* of 203 as the most predominant peak, and both followed by similar CH_2_ losses (*m/z* = 14). Additionally, a single water loss ion (*m/z* = 437), along with additional loss of butenyl is observed (*m/z* = 367).

**Niduchimaeralin B** was expected to be the reverse construction of niduchimaeralin A, i.e. the niduporthin PK backbone with phenylalanine incorporated. When comparing the fragmentation pattern to that of niduporthin similar fragments are observed. The major fragment is a peak with an m/z of 175, followed by fragments matching losses of CH_2_. Similarly to niduchimaeralin A water loss is also observed as a major fragment.
