# Supplementary material for: Linker Flexibility Facilitates Module Exchange in Fungal Hybrid PKS-NRPS Engineering
Source: PLoS One. 2016 Aug 23;11(8):e0161199. doi: 10.1371/journal.pone.0161199 (PMC4994942; doi:10.1371/journal.pone.0161199)
Supplement: S2 Text — (DOCX) [file pone.0161199.s012.docx]

# S2 Text. NMR structural elucidations of niduclavin and niduporthin

The structures of niduclavin and niduporthin were established based on interpretations of 1D and 2D NMR data (^1^H-NMR, ^13^C-NMR, DQF-COSY, edHSQC, HMBC, H2BC, and NOESY).

## Niduclavin

The DQF-COSY revealed five aromatic protons (H-5’ to H-7’), expected to originate from phenylalanine (C-1’ to C7’ and NH), and a methyl substituted cyclic octaketide (C1 to C-16, 6-CH_3_, 8-CH_3_, and 14-CH_3_). HMBC and DQF-COSY revealed a double bond between the α and β positions (C-2’ and C-3’) in the amino acid part of the molecule. The polyketide part of the molecule, was found to contain two diastereotopic CH_2_-groups (C-7 and C-9), once again indicating a cyclic molecule. Four CH_3_-groups (6-CH_3_, 8-CH_3_, 14-CH_3_, and 16) and five CH-groups (C-4 to C-6, C8, C-10 to C-13, and C-15) were also identified. Analysis of the DQF-COSY and HMBC data established a decalin ring system including the linking of all four CH_3_-groups to this part of the molecule. Furthermore, a W-coupling between H-15 and 14-CH_3_ indicated the double bond between C-14 and C-15 to be in an *E*-configuration.
Assignment of relative stereochemistry of niduclavin was hindered by two protons with the same chemical shift (H-5 and H-9a, both at δ 1.75 ppm). As a result, it was not possible to assign the relative stereo chemistry to the two methyl groups (6-CH_3_ and 8-CH_3_).

## Niduporthin

Downfield proton signals at 7-8 ppm were confirmed to belong to an indole heterocycle consisting of atoms C1’-C11’ as well as NH-1 and NH-2, confirming the expected incorporation of a tryptophan moiety. However, HMBC and DQF-COSY also revealed that the link between the α and β positions (C-2’ and C-3’) had been converted into a double bond, a modification speculated to be the result of endogenous *A. nidulans* enzymes. Secondly, DQF-COSY and HMBC could be used to identify an octaketide (C-1 to C-16), containing two CH_3_-groups (C-6 and C-16), three diastereotopic CH_2_-groups (C-7, C-8 and C-9) and five CH-groups (C-4 to C-6 and C-10 to C-15). Considering the size of the spin system, as well as the placement of the two olefinic protons, H-11 and H-12, a decalin ring system formed via a [4+2] cycloaddition was proposed, also confirmed by the H2BC data. Two carbonyls were identified; one belonging to a ketone (C-3), and one to a tetramic acid derived lactam (C-1), both originating from the polyketide part of the molecule. HMBC showed correlations to the ketone (C-3) from both the decalin ring (H-4) and from the tetramic acid (H-1’), thereby linking the two parts of the molecule together.
The relative stereochemistry could be assigned based on the NOESY experiments, as well as biosynthetic/mechanistic considerations. NOESY correlations were observed between protons H-6 and H-13, and H-4 and H-10. This fits with the hypothesis that the decalin ring is expected to be formed via a [4+2] cycloaddition, leading to location of protons H-10 and H-13 on the same face, and the protons H-5 and H-10 placed on opposite face of the decalin ring respectively, due to the cycloaddition being intramolecular.
